# Dual role of histone variant H3.3B in spermatogenesis: positive regulation of piRNA transcription and implication in X-chromosome inactivation

**DOI:** 10.1093/nar/gkac541

**Published:** 2022-06-29

**Authors:** Emeline Fontaine, Christophe Papin, Guillaume Martinez, Stéphanie Le Gras, Roland Abi Nahed, Patrick Héry, Thierry Buchou, Khalid Ouararhni, Bertrand Favier, Thierry Gautier, Jamal S M Sabir, Matthieu Gerard, Jan Bednar, Christophe Arnoult, Stefan Dimitrov, Ali Hamiche

**Affiliations:** Université Grenoble Alpes, CNRS UMR 5309, INSERM U1209, Institute for Advanced Biosciences (IAB), Site Sante′ – Allée des Alpes, La Tronche 38700, France; Institut de Génétique et Biologie Moléculaire et Cellulaire (IGBMC)/Université de Strasbourg/ CNRS/INSERM, 67404 Illkirch Cedex, France; Université Grenoble Alpes, CNRS UMR 5309, INSERM U1209, Institute for Advanced Biosciences (IAB), Site Sante′ – Allée des Alpes, La Tronche 38700, France; Institut de Génétique et Biologie Moléculaire et Cellulaire (IGBMC)/Université de Strasbourg/ CNRS/INSERM, 67404 Illkirch Cedex, France; Université Grenoble Alpes, CNRS UMR 5309, INSERM U1209, Institute for Advanced Biosciences (IAB), Site Sante′ – Allée des Alpes, La Tronche 38700, France; Institute for Integrative Biology of the Cell (I2BC), CEA, CNRS, Univ. Paris-Sud, Université Paris-Saclay, Gif-sur-Yvette 91198, France; Université Grenoble Alpes, CNRS UMR 5309, INSERM U1209, Institute for Advanced Biosciences (IAB), Site Sante′ – Allée des Alpes, La Tronche 38700, France; Institut de Génétique et Biologie Moléculaire et Cellulaire (IGBMC)/Université de Strasbourg/ CNRS/INSERM, 67404 Illkirch Cedex, France; Université de Grenoble Alpes, Etablissement Français du Sang, EA 7408, BP35, 38701 La Tronche, France; Université Grenoble Alpes, CNRS UMR 5309, INSERM U1209, Institute for Advanced Biosciences (IAB), Site Sante′ – Allée des Alpes, La Tronche 38700, France; Centre of Excellence in Bionanoscience Research, King Abdulaziz University, Jeddah 21589, Saudi Arabia; Institute for Integrative Biology of the Cell (I2BC), CEA, CNRS, Univ. Paris-Sud, Université Paris-Saclay, Gif-sur-Yvette 91198, France; Université Grenoble Alpes, CNRS UMR 5309, INSERM U1209, Institute for Advanced Biosciences (IAB), Site Sante′ – Allée des Alpes, La Tronche 38700, France; Université Grenoble Alpes, CNRS UMR 5309, INSERM U1209, Institute for Advanced Biosciences (IAB), Site Sante′ – Allée des Alpes, La Tronche 38700, France; Université Grenoble Alpes, CNRS UMR 5309, INSERM U1209, Institute for Advanced Biosciences (IAB), Site Sante′ – Allée des Alpes, La Tronche 38700, France; “Roumen Tsanev” Institute of Molecular Biology, Bulgarian Academy of Sciences, Sofia, Bulgaria; Izmir Biomedicine and Genome Center, Dokuz Eylul University Health Campus, Izmir 35330, Turkey; Institut de Génétique et Biologie Moléculaire et Cellulaire (IGBMC)/Université de Strasbourg/ CNRS/INSERM, 67404 Illkirch Cedex, France; Centre of Excellence in Bionanoscience Research, King Abdulaziz University, Jeddah 21589, Saudi Arabia

## Abstract

The histone variant H3.3 is encoded by two distinct genes, *H3f3a* and *H3f3b*, exhibiting identical amino-acid sequence. H3.3 is required for spermatogenesis, but the molecular mechanism of its spermatogenic function remains obscure. Here, we have studied the role of each one of H3.3A and H3.3B proteins in spermatogenesis. We have generated transgenic conditional knock-out/knock-in (cKO/KI) epitope-tagged FLAG-FLAG-HA-H3.3B (H3.3B^HA^) and FLAG-FLAG-HA-H3.3A (H3.3A^HA^) mouse lines. We show that H3.3B, but not H3.3A, is required for spermatogenesis and male fertility. Analysis of the molecular mechanism unveils that the absence of H3.3B led to alterations in the meiotic/post-meiotic transition. Genome-wide RNA-seq reveals that the depletion of H3.3B in meiotic cells is associated with increased expression of the whole sex X and Y chromosomes as well as of both RLTR10B and RLTR10B2 retrotransposons. In contrast, the absence of H3.3B resulted in down-regulation of the expression of piRNA clusters. ChIP-seq experiments uncover that RLTR10B and RLTR10B2 retrotransposons, the whole sex chromosomes and the piRNA clusters are markedly enriched of H3.3. Taken together, our data dissect the molecular mechanism of H3.3B functions during spermatogenesis and demonstrate that H3.3B, depending on its chromatin localization, is involved in either up-regulation or down-regulation of expression of defined large chromatin regions.

## INTRODUCTION

DNA in the eukaryotic nucleus is packaged into chromatin. Chromatin exhibits repeating structure and its basic unit, the nucleosome core particle, consists of an octamer of core histones (two of each H2A, H2B, H3 and H4) around which DNA is wrapped into ∼167 helical turns (1). This arrangement allows not only DNA packaging but also the control of all nuclear processes. Transcription is a dynamic process that requires permanent adjustments. To answer to these critical needs, the cell is using several epigenetic strategies, including chromatin remodeling, histone post-translational modifications and replacement of canonical histones with histone variants. This confers novel structural and functional properties of the nucleosomes and in turn, permits to either activate or repress transcription (2,3).

Histone variants are non-allelic isoforms of conventional histones. Each conventional histone family, except H4, has histone variants (4). Histone variants are incorporated into chromatin by dedicated histone chaperones (5–10). The histone H3 family, in addition to conventional H3, comprises two main histone variants, CENP-A and H3.3. H3.3 is encoded by two distinct genes *H3f3a* and *H3f3b*, which are localized on different chromosomes and show various organization, but the coded proteins exhibit identical amino acid sequence (11). H3.3 differs of only four amino acids residues from conventional H3 (12) and its deposition in chromatin is assisted by two specific chaperones, DAXX and ubinuclein (10,13).

The available data suggest that H3.3 is implicated in several essential nuclear events, including transcription and mitosis (9,14–16). Genome-wide ChIP analysis has shown that H3.3 is located on promoters, gene bodies and both centromeric and telomeric chromatin (9,14,16), but how H3.3 acts on these elements is not clear. The turnover of H3.3 depends on its localization, suggesting that the residence time of H3.3 association with specific chromatin loci is linked to its function (17).

H3.3 is also involved in spermatogenesis. A very strong phenotype, including severe infertility and reduced viability were observed in *Drosophila* loss of functions mutants, where both *H3.3A* and *H3.3B* were deleted by P element transposition (18). Inactivation of both *H3f3a* and *H3f3b* genes in mice led to early embryonic lethality and double *H3f3a*^KO/WT^/*H3f3b*^KO^ mutant males are totally infertile, while double *H3f3a*^KO/WT^/*H3f3b*^KO^ females are fertile, revealing a specific role of H3.3 in spermatogenesis (16). Another study showed that H3.3B depletion is alone sufficient to generate infertility in mice (19). Other reports claimed that *H3f3b* heterozygotes were growth-deficient with males being sterile because of problems at the level of round spermatids, while H3.3A depleted males were found subfertile with dysmorphic spermatozoa (20). A hypomorphic gene-trap mutation in *H3f3a* led to high mortality by weaning and the surviving animals exhibited severe infertility (21). Taken as a whole, these data demonstrate that H3.3 is an important player in spermatogenesis. The various genetic background of the animals used could explain the observed different impact of H3.3A and H3.3B on the progress of spermatogenesis in the different studies (discussed in (20)). The molecular mechanism of H3.3 functions during spermatogenesis and in particular, the impact of the H3.3 deficiency on transcription at genome-wide level, are, however, only superficially addressed.

Repression of the transcription of transposable elements (22,23), including long terminal repeats (LTR), long interspersed nuclear elements (LINE), and short interspersed nuclear elements (SINE) (Mouse Genome Sequencing Consortium 2002) are of prime importance. In germ cells, transposable elements, if transcribed, could spread into the host genome and pose an ongoing genetic threat. Hosts have developed specific strategies to silence them (24). For instance, RLTR10B containing retrotransposons such as MMERVK10C, one of the youngest retrotransposons in the mouse genome, was reported to be strongly down-regulated during spermatogenesis (25), through Trim33, a protein exhibiting ubiquitin ligase activity. No other chromatin associated proteins acting as repressors of RLTR10B were identified to date.

Another important sophisticated strategy for safeguarding the germ line genome in animals is the piwi (piRNA) pathway (24,26). This small RNA pathway is employed by the host animals to silence retrotransposons both transcriptionally and post-transcriptionally. This pathway uses complexes of the Argonaute subfamilies with piRNA to destroy the retrotransposon transcripts (26). Of note, the piwi pathway is also able to affect the epigenetic properties of the cognate genetic loci.

Here, we have studied the role of each one of H3.3A and H3.3B proteins in spermatogenesis by using conditional knock-out/knock-in (cKO/KI) epitope-tagged H3.3A^HA^ and H3.3B^HA^ mouse lines. We showed that H3.3B is required for spermatogenesis. The use of genome-wide approaches detailed the intimate mechanism of H3.3B spermatogenic functions. Our results revealed that H3.3B has a complex ‘dual’ role in spermatogenesis. Indeed, H3.3 positively regulates piRNA expression, but it is also required for meiotic sex chromosome inactivation and transcriptional repression of RLTR10B and RLTR10B2 repetitive elements. Our data illustrated the ability of the cell to use the same histone variant to control two opposite processes, preservation of an active transcriptional state and its repression.

## MATERIALS AND METHODS

### Mouse strains

Both H3.3B^HA^ and H3.3A^HA^ mutant mouse lines were established at the Phenomin-iCS (Phenomin – Institut Clinique de la Souris, Illkirch, France; http://www.ics-mci.fr/en/). For the generation of the H3.3B^HA^ line, a 2 kb fragment encompassing exon 2, exon3 and a part of exon 4 was amplified by PCR (from 129S2/SvPas ES cells genomic DNA) and subcloned in an iCS proprietary vector. This iCS vector contains a *LoxP* site as well as a floxed and flipped Neomycin resistance cassette. A DNA element encoding the FLAG-FLAG-HA epitope sequence was inserted in frame with the N-terminus of H3.3B. The linearized construct was electroporated in 129S2/SvPas mouse embryonic stem (ES) cells. After selection, targeted clones were identified by PCR using external primers and further confirmed by Southern blot with 5′ and 3′ external probes. Two positive ES clones were injected into C57BL/6N blastocysts, and the male chimaeras derived gave germline transmission. The H3.3A^HA^ mutant mouse line constructed similarly to the H3.3B^HA^ one. However, for the construction of the targeting vector, a DNA fragment, encompassing exon 2 was used. Mice were housed in the mouse facility of the Plateforme de haute Technologie Animale (PHTA, Grenoble, France; agreement number C 38 516 10001, registered protocol no. 321 at ethical committee C2EA-12). Of note, the mice used in the experiments exhibit C57BL/6 genetic background, which does not display a subfertile phenotype.

Genomic DNA, isolated from tails, was used for genotyping with the following primers: *H3f3a* forward, 5′-TTTGCAGACGTTTCTAATTTCTACT-3′; reverse-1, 5′-ATATCGGATTCAACTAAAACATAAC-3′; reverse-2 5′-CAGAGACCTGCCTGCCTGCTG-3′ (p1, p2 and p3, respectively in Supplementary Figure S1a); *H3f3b* forward-1 5′- CTGCCCGTTCTGCTCGCCGATT-3′; *H3f3b* forward-2, 5′- TCCTCATTCTACCACATGTTCA-3′; reverse, 5′- TCAATCTAGGCCTAAGACCAAA-3′ (p4, p5 and p6, respectively in Supplementary Figure S1b).

### Mouse *in vitro* fertilization and embryo development

Mouse sperm, obtained by manual trituration of caudae epididymides in M2 (Sigma-Aldrich) for 10 min. Sperm were allowed to swim for 10 min at 37°C in 1 ml of M2 medium. Sperm were centrifuged at 300 g before washing with M16 medium (Sigma-Aldrich), and their concentration adjusted for further use. Sperm were then capacitated in M16 medium with 2% fatty acid-free BSA at 37°C in a 5% CO_2_ incubator for 45 min prior IVF.

Oocytes were collected from mature OF1 females, synchronized by exposure to 5 units of pregnant mare serum gonadotropin (PMSG) and 5 units of human chorionic gonadotropin (hCG). Washed sperm were introduced into droplets containing 25 to 50 oocytes. Oocytes were incubated with: 1.5 × 10^5^ to 5 × 10^5^ capacitated sperm/ml (37°C, 5% CO_2_) in M16 medium, and unbound sperm were washed away after 4 h incubation. The number of 2-cell embryos were scored 24 h after insemination and transferred in KSOM medium for further pre-implantation development. Blastocysts were scored at 3.5 days after fertilization.

### Computer-assisted motility analysis (CASA)

10 μl of sperm suspension in M2 was immediately placed onto an analysis chamber (Leja Products B.V., Netherlands) of 100 μm depth and kept at 37°C for microscopic quantitative study of sperm movement. Sperm motility parameters were measured at 37°C using a sperm analyzer (Hamilton Thorn Research, Beverley). A minimum of 100 motile spermatozoa was analyzed for each assay. Motile sperm were defined by VAP >1 and progressive sperm were defined by VAP >30 and STR >70.

### Measurement of sperm vitality

40 μl of sperm were mixed with 20 μl of eosin 1% diluted in NaCl 9/1000 and 20 μl of nigrosine 10% diluted in NaCl 9/1000. Sperm were then layered onto glass slide and dried. A minimum of 100 spermatozoa was analyzed for each assay.

### Sperm morphology analysis

10 μl of the semen sample was displayed over a slide and dried at room temperature for 10 min, then fixed in ether/ethanol 1:1 and stained with Harris-Schorr stain.

### Sperm DNA assessment

Chromomycin A3 staining: semen samples were washed twice with 5 ml of PBS 1× and fixed in a methanol/acetic acid (3:1 v/v) solution at 4°C for at least 30 min. Cells were spread on Superfrost© slides and air dried at room temperature overnight. Cells were then labeled using a 0.25 mg/ml chromomycin A3 (CMA3) solution in McIlvaine buffer (pH 7) for 20 min, and washed twice for 2 min with McIlvaine buffer. Sperm nuclei were counterstained with a 0.5 μg/ml Hoechst solution for 3 min, and washed in PBS 1× for 3 min before mounting with DAKO mounting media.

Aniline blue: semen samples were washed twice with 5 ml of PBS 1×, 10 μl were spread on a slide, allowed to air dry and then fixed with a 3% glutaraldehyde solution in PBS 1× for 30 min at room temperature. Slides were then incubated for 5 min in water, 10 min in 5% aniline blue diluted in 4% acetic acid solution, twice for 2 min in water, 2 min in 70%, 90% and 100% ethanol solutions and finally for 2 min in toluene.

### Germ cell purification

To prepare germ cells, the seminiferous tubules were initially treated with collagenase (1 mg/ml for 15 min at 35°C) and 3 times washed with ice-cold DMEM, supplemented with 0.5% BSA, 10 mM sodium butyrate. The pelleted material was re-suspended in the same solution and germs cells were dissociated upon pipetting. After washing in PBS 1× and centrifugation at 2500 g for 5 min, the cells were fixed in formalin overnight at 4°C and stocked in PBS 1× at 4°C.

### Immunohistology

Testes and epididymis were isolated from adult mice and were formalin-fixed overnight, dehydrated and paraffin-embedded. Ten serial paraffin sections (10 μm thick) of fixed mouse tissues were deparaffinized in toluene and hydrated through series of 10 min baths of 100%, 90%, 80%, 50% ethanol solution in PBS 1×. The sections were finally washed in PBS 1×. At this stage, the slides were used for staining with hematoxylin eosin (Sigma). For immunostaining, the sections were incubated for 40 min at 95°C in sodium citrate 10 mM pH 6 (Sigma), 0.05% Tween 20 (Carlo Erba) and blocked with 5% dried milk in PBS for 1 h at room temperature. The acrosomes were stained in red by incubation during 1 h at 37°C with lectin PNA alexa 568 (Life Science L32458) diluted 1/500. DNA was counterstained with the Hoechst 33342 (Invitrogen H3570). After washing in PBS 1×, the slides were mounted in fluorescent mounting medium (Dako). The sections were then observed with Zeiss Axio Imager Z1 microscope with a Plan-Apochromat 20×/0.8 M27 objective. Images were acquired with a Hamamatsu Orca Flash the camera with Zeiss Axiovision 4.8.10 software.

### Immunostaining on testis section

Immunostaining was carried out on deparaffinized testis sections (8μm), rehydrated in ethanol bath (100 to 30%) PBS 1× for 5 min and incubated for 40 min at 95°C in 10 mM sodium citrate pH 6 (Sigma), 0.05% Tween 20 (Carlo Erba). Next, they were permeabilized with 0.1% Triton-X at room temperature for 30 min. After blocking in 5% dried milk and washing in PBS 1×, the testis sections were incubated in 1% dried milk for 1 h at 37°C in a humidified chamber using the following antibodies: anti-PLZF 1/100 (Santa Cruz sc22839), anti-γH2AX 1/500 (Abcam ab81299), anti-H3.3 1/100 (Millipore 09-838). Anti-SCP3 (Santa Cruz sc20845), anti-lectin PNA 1/500 (ThermoFisher L32458) was incubated 3 h at 37°C in 1% milk with 1/100. After washes in PBS 1× for 30 min at room temperature, a secondary cyanine 2 (Jackson lab 115-225-146), cyanine 3 (Jackson lab 705–165-147-174-00) or cyanine 5 (Jackson lab 111-175-144) antibodies were applied for 30 min at room temperature in 1% fetal bovine serum, PBS 1×. All samples were incubated for another 5 min in Hoechst 33342 (Invitrogen H3570) to counterstain DNA.

### Immunostaining of germ cells

Germ cells were spread out on a slide, dried for 2 h at room temperature, rehydrated in PBS 1× for 5 min and incubated for 40 min at 95°C in 10 mM sodium citrate pH 6 (Sigma), 0.05% Tween 20 (Carlo Erba). Next, they were permeabilized with 0.1% Triton-X at room temperature for 30 min. After blocking in 5% dried milk and washing in PBS 1×, cells were incubated in 1% dried milk for 1 h at 37°C in a humidified chamber using the following antibodies: anti-HA 1/800 (Roche 1867423), anti-H3 1/200 (Millipore 05-928), anti-H4 1/200 (Abcam ab10158), anti-γH2AX 1/500 (Abcam ab81299), anti-H3K9me3 1/600 (Abcam ab8898), anti-H3.3 1/100 (Millipore 09-838). After washes in PBS 1× for 30 min at room temperature, a secondary cyanine 2 or cyanine 3 antibodies were applied for 30 min at room temperature in 1% fetal bovine serum, PBS 1×. All samples were incubated for another 5 min in Hoechst 33342 (Invitrogen H3570) to counterstain DNA.

Of note, immunostaining with the 1/100 CD9 (BD pharmagen 553758) antibody was performed immediately drying the germ cells for 2 h at room temperature and their rehydratation in PBS 1× for 5 min. The slides were then blocked with 10% bovine fetal serum for 1h at room temperature and subsequently incubated in humidified chamber with the antibody for 1 h at 37°C.

### Microscopy

Microscopy visualization was performed using a Zeiss Axio Imager Z1 microscope apotome module. The fixed germ cells were observed with a Plan-Apochromat ×63 objective and the Zeiss Axiocam camera. The meiotic and the post-meiotic cells were identified by their distinct nucleus morphology after DAPI staining (27,28). γH2AX staining was used for the identification of sex chromosomes (28,29). Germ stem cells were identified by CD9 antibody (30).

### TUNEL test

TUNEL test was carried out on both semen and deparaffinized testis sections. Semen samples were washed twice with 5 ml of PBS 1× and fixed in a methanol/acetic acid (3:1 v/v) solution at 4°C for at least 30 min. Cells were spread on Superfrost© slides and air dried at room temperature overnight. Cells and sections were permeabilized using 0.1% (v/v) Triton X-100, 0.1% (w/v) sodium citrate in PBS 1× for 2 min and labeled by terminal deoxynucleotidyl transferase mediated deoxy-UTP nick end labeling (TUNEL) according to the protocol provided with the *In Situ* Cell Detection Kit (Roche Diagnostic, Manheim, Germany). Nuclei were counterstained with 0.5 μg/ml Hoechst solution for 3 min, washed in PBS 1× for 3 min and mounted with DAKO mounting media. All testes section image were performed with Zeiss Axio Imager Z1 microscope with a Plan-Apochromat 20×/0.8 M27 objective. Images were acquired with a Hamamatsu Orca Flash the camera with Zeiss Axiovision 4.8.10 software and analyzed with Zen Office 2.3 lite. Cell type identification and tubule seminiferous stage were performed according to (27,31) in using serial section labeling with PNA-lectin.

### Fractionation of spermatogenic cells

The fractionation of the spermatogenic cells was carried out essentially by using the available protocol (32,33). Briefly, male mice were euthanized and testes were recovered. After the removal of albuginea, seminiferous tubules were incubated in a 1 mg/ml collagenase solution for 15 min at 35°C. After centrifugation, collagenase-treated cells were suspended in 0.5% BSA–DMEM medium. The aggregates were dissociated by rapid pipetting for 10 min and filtered through a 100 μm filter. The total germ cell suspension was loaded on the top of a 2–4% BSA gradient. The cells were let to sediment for 70 min. Cell fractions were collected and processed for examination with a phase contrast microscope. Meiotic and post-meiotic cell fractions (mainly round spermatids) were pooled and used for further analysis. The post-meiotic fraction contains only small contaminations of elongated spermatids.

### RT-qPCR

Reverse transcriptase quantitative real-time PCR (RT-qPCR) was used to measure RNA expression levels of the studied genes. Total RNA was isolated from seminiferous tubules of one mouse in 1 ml of TRIzol (Invitrogen) using Nucleospin RNA (Macherey-Nagel) according to the manufacturer's instructions. Reverse-transcribed was realized with iScript (Biorad) and random hexamer and poly-dT primer mix. *Rer1* was used as the internal reference gene. Supermix SYBR Green iTaq Universal (BioRad) and C1000 Touch Thermal Cycler (BioRad) real-time system were used. Primers used were as follows: *Rer1*-F, 5′GCCTTGGGAATTTACCACCT; *Rer1*-R, 5′-CTTCGAATGAAGGGACGAAA-3′; *H3f3a*-F, 5′-ACAAAAGCCGCTCGCAAGAG-3′; *H3f3a*-R, 5′-ATTTCTCGCACCAGACGCTG-3′; *H3f3b*-F, 5′-TGGCTCTGAGAGAGATCCGTCGTT-3′; *H3f3b*-R, 5′-GGATGTCTTTGGGCATGATGGTGAC-3′; *Prm1*-F, 5′-ATGCTGCCGCAGCAAAAGCA-3′; *Prm1*-R, 5′-CACCTTATGGTGTATGAGCG-3′; *Prm2*-F, 5′-ATGGTTCGCTACCGAATGAG-3′; *Prm2*-R, 5′-TTAGTGATGGTGCCTCCTAC-3′; *Tnp1*-F, 5′-ATGTCGACCAGCCGCAAGCT-3′; *Tnp1*-R, 5′-TCACAAGTGGGATCGGTAAT-3′; Abcg8-F, 5′-GATTTCCAATGACTTCCGGGAC-3′; Abcg8-R, 5′-AGTGACATTTGGAGACGACATC-3′; Ptpro-F, 5-TCCATGAACGAAGAGGAAGGAG-3′; Ptpro-R, 5′-TTGAGTGAGCATGACGATGATG-3′; Fmnl1-F, 5′-CATGAACACACTCACCTTCCTG-3′; Fmnl1-R, 5′- CTCTGAAGTCGGAAACCATAGG-3′; Hand2-F, 5′-CCAAACTCTCCAAGATCAAGAC-3′; Hand2-R, 5′-TTGTCGTTGCTGCTCACTGTG-3′; Vnn1-F, 5′-ACTCCATCTATGTTGTGGCGAAC-3′; Vnn1-R, 5′-GGACATTGAACTGATCTTCTCC-3′; Zfp811-F, 5′-GCTGGTGACCTTCGAGGATG-3′; Zfp811-R, 5′-CTTATGTCTCCTATGGAAACCAC-3′.

### Western blot

Germ cells were isolated from seminiferous tubules or purified meiotic and post-meiotic cells and collected in 1 ml TRIzol (Life Technologies). After protein extraction with TRIzol protocol, 20 μl of sample HA flag was loaded and separated on 15% SDS-PAGE. Proteins were detected using anti-H3 1/5000 (Millipore 05–928) and anti-HA 1/2500 (Abcam ab9110). The western blot with sample *H3f3a^WT^*, *H3f3a^KO^* and *H3f3b^KO^* in whole testis or purified meiotic and post-meiotic cells realized on mini-PROTEAN TGX stain free 4–20% (BioRad). Total proteins were visualize by UV activation for 1 min. The quantity of H3.3 protein detected by anti-H3.3 (Millipore 09-838). The Western blot was carried out as described (34,35).

### Native ChIP-seq

#### Micrococcal nuclease preparation and double-immunoaffinity purification of native FLAG-HA tagged mononucleosomes

Meiotic and post-meiotic cells expressing FLAG-HA tagged endogenous H3.3 were lysed in hypotonic buffer (10 mM Tris–HCl at pH 7.65, 1.5 mM MgCl_2_, 10 mM KCl) and disrupted by Dounce homogenizer. The cytosolic fraction was separated from the nuclei by centrifugation at 4°C. Nuclei were re-suspended in sucrose buffer (20 mM Tris–HCl at pH 7.65, 15 mM KCl 60 mM NaCl, 0.15 mM spermine, 0.5 mM spermidine) adjusted with high-salt buffer (20 mM Tris–HCl at pH 7.65, 25% glycerol, 1.5 mM MgCl_2_, 0.2 mM EDTA, 900 mM NaCl) to get a final NaCl concentration of 300 mM. The nuclear-soluble fraction was recovered by centrifugation at 4°C. The pellet containing chromatin fraction was incubated in sucrose buffer containing 1 mM CaCl_2_, and MNase (2.5 U/g of cells) 10 min at 37°C. Digestion was stopped with 4 mM EDTA and mononucleosomes were recovered by centrifugation at 4°C. Tagged nucleosomes were immunoprecipitated with anti-FLAG M2-agarose (Sigma), eluted with FLAG peptide (0.5 mg/ml), further affinity-purified with anti-HA antibody-conjugated agarose, and eluted with HA peptide (1 mg/ml). The HA and FLAG peptides were first buffered with 50 mM Tris–Cl (pH 8.5), then diluted to 4 mg/ml in TGEN 150 buffer (20 mM Tris at pH 7.65, 150 mM NaCl, 3 mM MgCl_2_, 0.1 mM EDTA, 10% glycerol, 0 0.01% NP40), and stored at −20°C until use. Between each step, beads were washed in TGEN 150 buffer. Nucleosomes were submitted to RNase and proteinase K digestion, and DNA was extracted by phenolchloroform.

#### Library construction and sequencing

Libraries were prepared using the Diagenode MicroPlex Library Preparation kit version v.2 02.15, and sequenced on Illumina Hiseq 4000 sequencer as single-end 50 bp reads following Illumina's instructions. Image analysis and base calling were performed using RTA 2.7.3 and and bcl2fastq 2.17.1.14. Adapter dimer reads were removed using DimerRemover v0.9.2 (https://sourceforge.net/projects/dimerremover/). Reads were mapped to the mouse genome (mm9) using Bowtie (36) v1.0.0 with the following arguments : -m 1 –strata –best -y -S –l 40 -p 8.

### MeDIP-seq and hMeDIP-seq

DNA immunoprecipitation, librairies construction and sequencing were done as previously described (37).

### Computational analyses of ChIP-seq and DIP-seq datasets

Heatmaps and quantifications of the ChIP-seq and DIP-seq data were performed running seqMINER (38), using datasets normalized to 10 million uniquely mapped reads. As reference coordinates, we used the Ensembl 67 database (for coding genes) of the mouse genome (mm9), the annotated RepeatMasker (RMSK) database (for DNA repeats) and the piRNA cluster database generated by the small RNA group Mainz University (http://www.smallrnagroup.uni-mainz.de/piRNAclusterDB.html) after having translated mm10 coordinates to mm9 using liftOver (UCSC: https://genome.ucsc.edu/cgi-bin/hgLiftOver). For chromosome coverage analysis, the number of reads for each non-overlapping 40 kb windows of the Mouse genome (mm9/NCBI37) was computed for each sample using BEDtools v2.26 (39). Then, data were normalized to 10 million reads and plots were generated using custom R scripts.

### RNA-seq

#### Library construction and sequencing

After isolation of total cellular RNA from WT and *H3f3b^KO^* meiotic and post-meiotic cells, libraries of template molecules suitable for strand specific high throughput DNA sequencing were created using ‘TruSeq Stranded Total RNA Sample Preparation Guide’ (PN 15031048). The libraries were sequenced on Illumina Hiseq 4000 sequencer as single-end 50 base reads following Illumina's instructions. Image analysis and base calling were performed using RTA 2.7.3 and and bcl2fastq 2.17.1.14. Adapter dimer reads were removed using DimerRemover. (https://sourceforge.net/projects/dimerremover/). Reads were mapped onto the mm9 assembly of the mouse genome by using Tophat version: tophat-2.0.14 (39) and the bowtie version bowtie-2–2.1.0 (36). Only uniquely aligned reads have been retained for further analyses.

#### Gene expression analysis

Quantification of gene expression has been performed using HTSeq-0.6.1 (http://www-huber.embl.de/users/anders/HTSeq) and gene annotations from Ensembl release 67. Read counts were normalized across libraries with the method proposed by Anders and Huber (40). Comparisons of interest were performed using the method proposed by (41) implemented in the DESeq2 Bioconductor library (DESeq2 v1.14.1). Independent filtering based on the mean of normalized counts was performed in order to filter out those genes that have no or little chance of showing significance evidence of differential expression (without looking at their statistic). Indeed, genes with very low counts are not likely to be significantly differentially expressed typically due to high dispersion. This independent filtering results in increased detection power. Genes with high Cook's distance were also filtered out. Cook's distance is a measure of how much a single sample is influencing the fitted coefficients for a gene, and a large value of Cook's distance is intended to indicate an outlier count. Resulting *P*-values were adjusted for multiple testing using the Benjamini and Hochberg method (42). The following thresholds have been used to select significantly differentially expressed genes:

- *P*-value adjusted for multiple testing < 0.01
- |log_2_ Fold-Change| > 1

#### piRNA cluster expression analysis

Using IntersectBed from BEDTools release v2.26.0, split-mapped reads and reads that overlap genes annotated by Ensembl were removed (IG_C_gene, IG_D_gene, IG_J_gene, IG_V_gene, Mt_rRNA, Mt_tRNA, polymorphic_ pseudogene, protein_coding, pseudogene, rRNA). The region corresponding to those genes was extended to 3kb upstream of the transcription start site and 10 kb downstream of the transcript end site, in order to minimize signal from polymerase read-through from genic transcripts. The overlap was performed on the opposite strand as the library preparation protocol used to construct these RNA-seq libraries leads to sequence the strand generated during first strand cDNA synthesis. Quantification of piRNA cluster expression has been performed using IntersectBed from BEDTools release v2.26.0q. piRNA read counts were added to those of gene expression's and used as follows: read counts were normalized across libraries with the method proposed by Anders and Huber (40). Comparisons of interest were performed using the method proposed by Love *et al.* (41) implemented in the DESeq2 Bioconductor library (DESeq2 v1.14.1). The following thresholds have been used to select significantly differentially expressed piRNA clusters:

- *P*-value adjusted for multiple testing < 0.01
- |log_2_ Fold-Change| > 0.5

#### DNA repeat expression analysis

Repeat analysis was performed following the methodology described in Papin *et al.* (23). Data were normalized based upon library size. Significance of the difference of repeat read counts between RNA samples was assessed using the Bionconductor package DESeq. To avoid over- or underestimating fold enrichments due to low sequence representation, repeat families with less than 5 RPM (reads per million mapped reads) per RNA sample were excluded from further analysis. The following thresholds have been used to select significantly differentially expressed DNA repeats:

- *P*-value adjusted for multiple testing < 0.01
- |log_2_ Fold-Change| > 0.5

## RESULTS

### *H3f3b* null male mice are infertile

**Figure 1.**
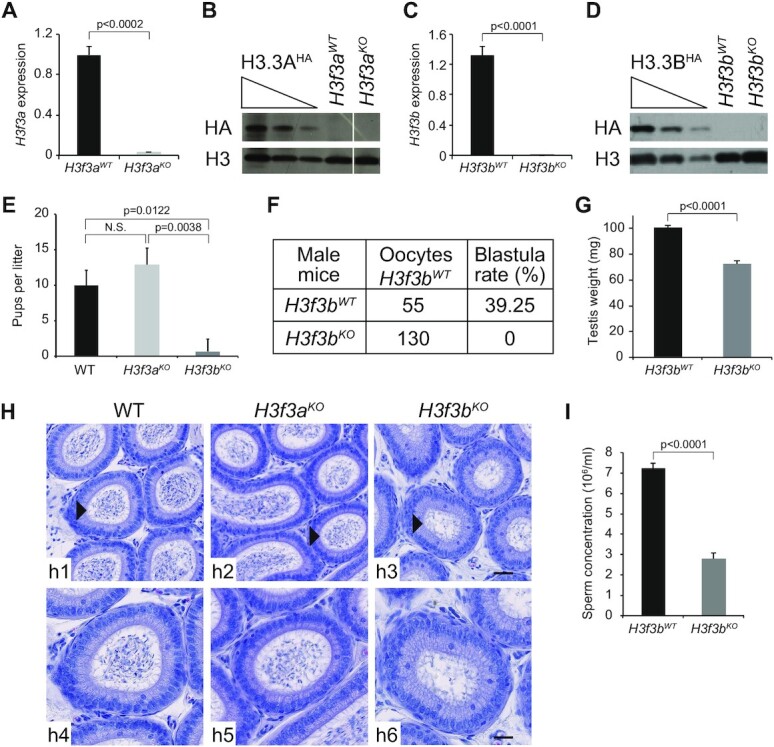
*H3f3b^KO^*, but not *H3f3a^KO^* mice, exhibit decreased sperm production and severe infertility. (**A–D**) Efficient depletion of H3.3A and H3.3B in the testis of the indicated knock-out mice. qPCR measurement of the mRNA level (A, C) and Western blotting (anti-HA) analyses (B, D) of either H3.3A (A, B) or H3.3B (C, D). (A, C) The average values of the mRNA of three biologically independent experiments are shown. (B, D) Total cellular lysates derivedfrom testis extracted from H3f3a^HA^, H3f3a WT and H3f3a KO mice (B) or H3f3b^HA^, H3f3b WT and H3f3b KO mice (D) were submitted to Western blot analysis. (**E**) Numbers of pups per female OF1 mated with either *H3f3b^WT^*, *H3f3a* null or *H3f3b* null male. The average of 9 litters is presented for 3 males by genotype. (**F**) Number of 2-cell and blastocysts obtained by *in vitro* fertilization using sperm from either control or *H3f3b* null males and oocytes prepared from control *H3f3b^WT^* females. (**G**) Testis weight for control and *H3f3b* null males. The average of four testes of different genotypes was presented. (**H**) Hematoxylin-stained cross-sections of epididymis of control (h1, h4), *H3f3a^KO^* (h2, h5) and *H3f3b^KO^* (h3, h6) male mice; h4–h6 show the enlarged epididymis cross-sections indicated by an arrow head in h1–h3; scale bars, 30 μm (h3) and 70 μm (h6). (**I**) Sperm concentration (millions per milliliter) for control *H3f3b^WT^* (*n* = 5) and *H3f3b* null males (*n* = 2). (A, C, E, G, I) *P*-values were calculated from Student's unpaired *t*-tests.

The availability of transgenic conditional knock-out/knock-in (cKO/KI) epitope-tagged H3.3A^HA^ and H3.3B^HA^ mouse lines allowed, by using homologous recombination, to produce either *H3f3a* or *H3f3b* null mice (see Supplementary Figure S1a–f and Method section for the gene targeting strategy). Both RT-qPCR and Western blotting showed that the proteins were absent from the respective mouse line (Figure 1A–D).

The male null *H3f3b* mice, mated with WT females, exhibited very severe subfertility problems in contrast to *H3f3a* null mice, which were found as fertile as the WT mice (Figure 1E). In agreement, *in vitro* fertilization using null *H3f3b* mouse spermatozoa failed completely (Figure 1F). The testis weight of the *H3f3b* null mice were ∼30% smaller than this of the control WT ones (Figure 1G). A decrease of the number of spermatozoa in the cauda epididymis of *H3f3b* null mice was also observed (Figure 1H). Accordingly, the concentration of sperm in the *H3f3b^KO^* animals was found to be only ∼40% compared to this of the WT animals (Figure 1I). The vitality of the mature spermatozoa from the *H3f3b* null mice was slightly decreased (Supplementary Figure S1g), but they showed increased level of DNA fragmentation as judged by the TUNEL assay (Supplementary Figure S1h) and markedly altered morphology (Supplementary Figure S1i), including both abnormal tails and heads (Supplementary Figure S1j–m). In addition, strong alterations of DNA compaction in the spermatozoa from *H3f3b* null mice were observed (Supplementary Figure S1n), that presumably reflects the abnormal exchange of histone to protamines (Supplementary Figure S1o). However, our data suggested that the absence of H3.3B did not affect the expression level of both transition proteins (TPs) (Supplementary Figure S1p) and protamines (Supplementary Figure S1q,r), unveiling that this is very likely not due to a decrease of the expression of these proteins. Taken collectively, these results reveal, in agreement with the reported data (19) (where mice with very similar background to this of our mouse lines were used), that H3.3B, but not H3.3A, is essential for male fertility and, in particular, for functional spermatozoa formation.

### The absence of H3.3B is associated with strong decrease of the amount of post-meiotic cells

**Figure 2.**
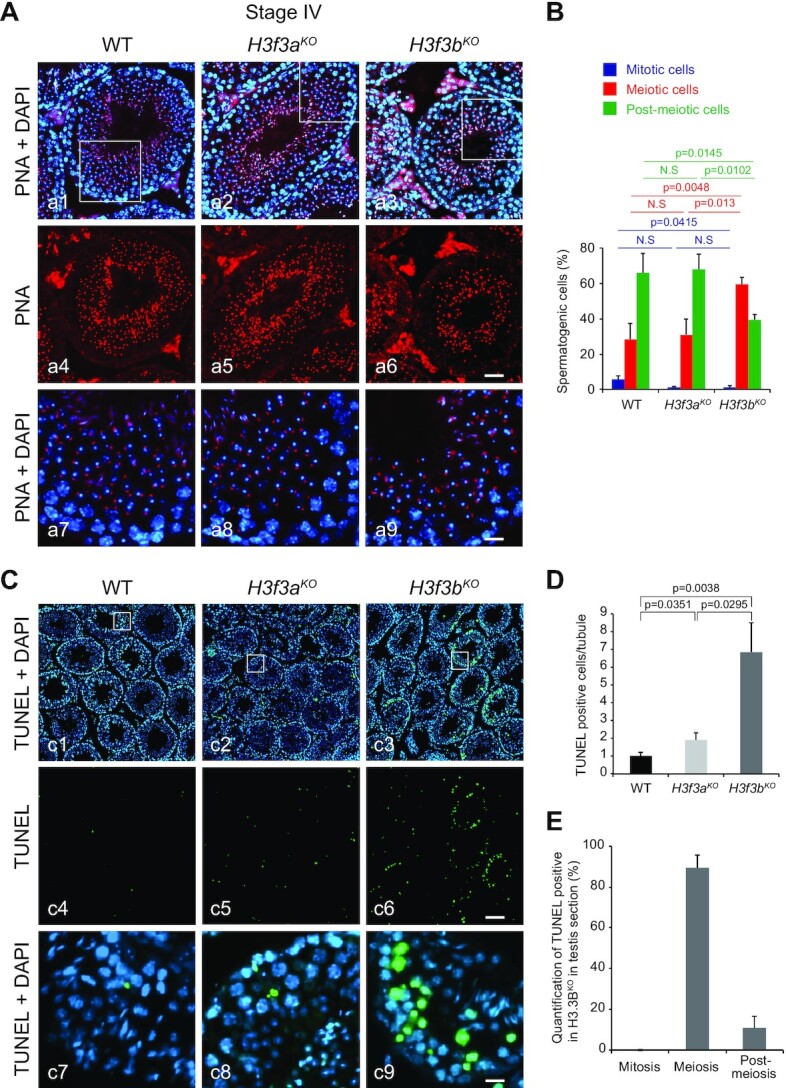
The absence of H3.3B leads to strong alterations in the meiotic/post-meiotic transition. (**A**) WT, *H3f3a^KO^* and *H3f3b^KO^* stage IV testis sections stained with DAPI (blue) for nucleus detection and with PNA (Peanut Agglutinin, red) for acrosome visualization. a7–a9 correspond to the enlargement of the white square in a1–a3. Scale bars, 70 μm (a6) and 15 μm (a9). (**B**) Quantification of the different types of spermatogenic cells in the indicated genotypes. In post-meiotic cell quantification included round spermatids only and not elongated spermatids and spermatozoa. (**C**) TUNEL staining (green) of testis sections from control, *H3f3a* and *H3f3b* null males; blue, DAPI staining. c7–c9 correspond to the enlargement of the white square in c1–c3. Scale bars, 70 μm (c6) and 10 μm (c9). (**D**) Normalized number of TUNEL positive cells per seminiferous tube for control *H3f3b^WT^* (*n* = 3), *H3f3a^KO^* (*n* = 2) and *H3f3b^KO^* (*n* = 3) males. (**E**) Distribution of TUNEL positive spermatogenic cells from *H3f3b^KO^* male according to their maturation state. (B, D, E) *P*-values were calculated from Student's unpaired *t*-tests.

To shed light on the origins of the alterations in the morphology and in the dramatic decrease of produced spermatozoa in the *H3f3b^KO^* animals, we next sought to identify the testis cell types that are affected by the absence of H3.3B. To this end, we analyzed the different testis cell types in WT, *H3f3a^KO^* and *H3f3b^KO^* mice. For simplicity, in our analysis we grouped the cells in three different classes, namely mitotic, meiotic and post-meiotic cells. What is worthy to note is that, the very large majority of meiotic cells are pachytene spermatocytes and those of the post-meiotic ones are round spermatids (43,44). The absence of H3.3B resulted in dramatic decrease of the round spermatid quantity in the seminiferous tubules, whereas the absence of H3.3A did not affect the production of round spermatids (Figure 2A and Supplementary Figure S2a, b). Quantification of the different cell types in the testis of the *H3f3b^KO^* males showed that the ratio meiotic cells/post-meiotic cells is dramatically altered. Indeed, in both control and *H3f3a* null testis, the amount of meiotic cells is ∼2.5-fold smaller compared to the amount of post-meiotic cells, while in the *H3f3b* null testis, this is the opposite with meiotic cells being ∼1.5-fold more than post-meiotic cells (Figure 2B).

Since H3.3 is involved in cell division (15), the simplest explanation of this phenomenon would be some implication of H3.3B in meiosis. If this is correct, one should expect the absence of H3.3B, but not of H3.3A, to affect the meiotic process and to generate defects that should lead to apoptotic outcomes of the meiotic cells and consequently, to the production of smaller amount of post-meiotic cells. With this in mind, we have used the TUNEL assay to visualize DNA fragmentation in the cells of the seminiferous tubules of the three types of mice. As seen (Figure 2C), the amount of TUNEL positive cells in the seminiferous tubules of the *H3f3b^KO^* animals is much higher compared to this of both WT and *H3f3a^KO^* animals. The quantification showed that the absence of H3.3B resulted in 7-fold increase of TUNEL positive cells in the seminiferous tubules of the *H3f3b^KO^* mice (Figure 2D). Importantly, more than 85% of the TUNEL positive cells belonged to the meiotic pool (Figure 2E). We concluded that H3.3B is, as hypothesized, involved in meiosis and that its absence led to a depletion of post-meiotic cells.

We next examined the number of spermatogonia in the seminiferous tubules of the *H3f3b^KO^* mice using the PLZF germline stem cells marker. Loss of *H3f3b* did not affect spermatogonia formation or proliferation (Supplementary Figure S2c, d), but led to progressive depletion of post-meiotic cells and finally caused infertility, indicating its critical role in meiosis.

### In middle pachytene H3.3B appears after γH2AX on sex chromosomes

**Figure 3.**
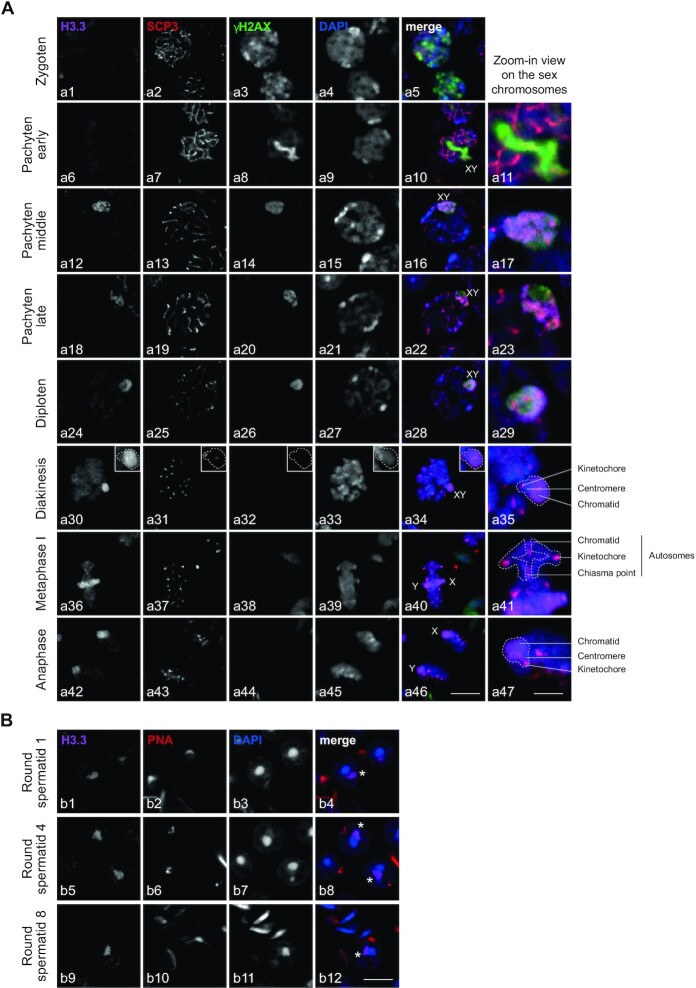
H3.3B localized on the sex chromosomes from the middle pachytene to round spermatids during spermatogenesis. (A, B) Immunostaining of spermatogenic cells from meiosis (**A**) to post-meiosis (**B**). Cells were triple-stained on WT testis section with anti-H3.3, anti-γH2AX, anti-SCP3 (A) or anti-PNA (B) as well as DAPI. Developmental stages were classified by staining profiles of SCP3, γH2AX and PNA. Scale bars, 8 μm (a46), 3 μm (a47) and 8 μm (b12). The asterix represents the sex chromosome either x or y (b4, b8, b12).

To deeply investigate the role of *H3f3b* in spermatogenesis, we performed immunostaining analysis of H3.3b distribution in spermatocytes from meiosis (Figure 3A), to post-meiosis (Figure 3B). Developmental stages were classified by staining profiles with anti-SCP3/γH2AX in meiosis and PNA labeling in post-meiosis and according to the description of the different stages of spermatogenesis (according to Meistrich and Hess (27)). At the zygoten stage, we observed the absence of H3.3 (Figure 3A, panel a1). Cells at the zygoten stage are easily identifiable since they have a small nucleus and diffuse γH2AX labeling in the nucleus (Figure 3A, panel a3) (45). In addition, SCP3 labeling (Figure 3A, panel a2) appears as an entangled line, a characteristic image corresponding to the labeling of the synaptonemal complex. This complex links together sister chromatids of the same chromosome until the pachytene late stage (Figure 3A, panel a19) (45). Between all meiotic stages (Figure 3A, panels a4, a9, a15, a21, a27) the volume of the cells grows progressively. The pachytene early stage is identifiable by typical γH2AX labeling only on the rod-shaped sex chromosomes (Figure 3A, panel a8). At this stage, H3.3 was totally absent from the whole cell (Figure 3A, panel a6), but appeared at the pachytene middle stage, when γH2AX labelled sex chromosome condense to form the XY body (Figure 3A, panels a12, a14) (46). At the pachytene late and diploten stages, H3.3 and γH2AX remained on the XY body (Figure 3A, panels a18, a20, a24, a26) and showed similar labeling (Figure 3A, panels a17, a23, a29) (47).

Diploten cells were easily identified as they showed a large cell volume (Figure 3A, panel a27) and sparse SCP3 labeling (Figure 3A, panel a25) along the synaptonemal complex. The destruction of the synaptonemal complex starts at the diploten stage with the disappearance of SCP3 in diakinesis excepted at the kinetochore in the centromeric region (Figure 3A, panels a31, a35). Chromosomes remained linked by chiasmas (area of chromatid overlap) (Figure 3A, panel a41). At this stage, there was a separation of homologous chromosomes that migrate to the centrosomes during the first meiotic division (Figure 3A, panels a40, a46) (48,49). H3.3 remains enriched on sex chromosomes from diakinesis to anaphase I (Figure 3A, panels a30–a42). In metaphase II, SCP3 disappeared from the centromeric region. Kinetochores were called upon to separate sister chromatids. This step was extremely fast and only very few cells were visible.

In post-meiosis, H3.3 remains localized on the sex chromosomes of round spermatids (Figure 3b, panels b1, b5, b9) resulting from the meiotic divisions. Round spermatids were small, haploid, round-shaped cells with a centrally located area of round heterochromatin, named chromocenter (Figure 3B, panels b3, b7, b11) (28,50). The sex chromosomes were present in an area of facultative (light grey in DAPI panel) round heterochromatin juxtaposed to the chromocenter (Figure 3b, panels b3, b7, b11) named post-meiotic sex chromatin (PMSC) (51–53).

All together these data show that H3.3 appears on sex chromosomes in the middle pachytene after γH2AX and remain associated during all transition stages from meiosis to post-meiosis.

### H3.3B is implicated in transcriptional repression of sex chromosomes

**Figure 4.**
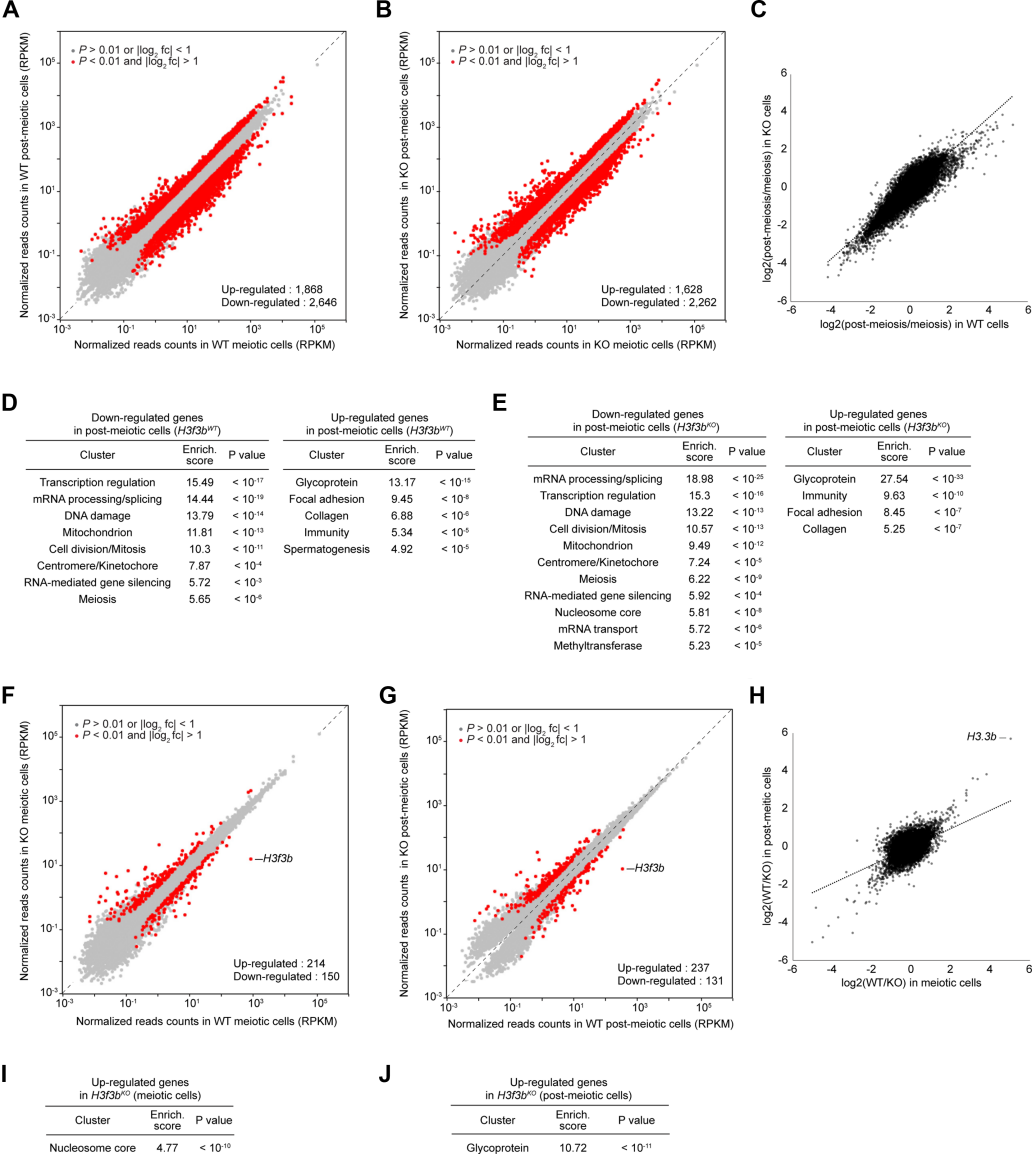
Genome-wide transcriptome analysis of meiotic and post-meiotic cells in the absence of H3.3B. (A, B) Scatter plots comparing gene expression profiles of WT post-meiotic and meiotic cells in the presence (**A**) or absence (**B**) of H3.3B. Red dots indicate differentially expressed genes. (**C**) Scatter plot of all pairwise log2-fold change in gene expression during meiotic to post-meiotic transition between WT and KO cells. (D, E) Functional annotation clustering of differentially expressed genes in post-meiotic cells in the presence (**D**) or absence (**E**) of H3.3B. (F, G) Scatter plots comparing gene expression profiles of *H3f3b^WT^* versus *H3f3b^KO^* in meiotic (**F**) and post-meiotic cells (**G**). Red dots indicate differentially expressed genes. (**H**) Scatter plot of all pairwise log_2_-fold change in gene expression in the absence of H3.3B between meiotic and post-meiotic cells. (I, J) Functional annotation clustering of up-regulated genes in meiotic (**I**) and post-meiotic cells (**J**) in the absence of H3.3B.

The absence of H3.3B could affect specific genes playing a key role in meiosis and this could in turn lead in severe dysfunction of meiotic cells and consequently to apoptosis and cell death. To test this, we have carried out genome-wide transcriptome studies. The comparative analysis of the meiotic/post-meiotic WT cell transcriptome showed, in agreement with the reported data (54), that the meiotic/post-meiotic transition is associated with vast transcriptional reprogramming, including thousands of up- and down-regulated genes, ranging in well-defined clusters (Figure 4A, D). The absence of H3.3B had only a slight effect on the number of deregulated genes and cluster types during the meiotic to post-meiotic transition (Figure 4B, E). Accordingly, genes are dysregulated in the same way during the meiotic/post-meiotic transition in both WT and KO cells (Figure 4C). This suggests that the H3.3B effect on the meiotic/post-meiotic transition could not be directly associated with alterations of the expression of specific genes, but rather reflects its implication in other process(es). Furthermore, only 214 and 150 genes were significantly up- and down-regulated, respectively, in the *H3f3b* null meiotic cells (Figure 4F, Supplementary Figure S3a and Supplementary Table S1). A single cluster, consisting of significantly up-regulated histone genes located on chromosome 13 was found (Figure 4I). In the case of H3.3B deficient post-meiotic cells, the picture was very similar (Figure 4G, H), but the single observed cluster consisted of genes coding for glycoproteins (Figure 4J, Supplementary Figure S3a and Table S2). This is in agreement with recent data, showing only a minor role of H3.3 in the control of transcription in both mouse embryonic fibroblast (MEFs) and mouse embryos (15,16). It is worth noting that the absence of H3.3B was reported to affect some genes involved in mouse spermatogenesis (19). However, in this latter case the cutoff used was only 1.5-fold (19).

**Figure 5.**
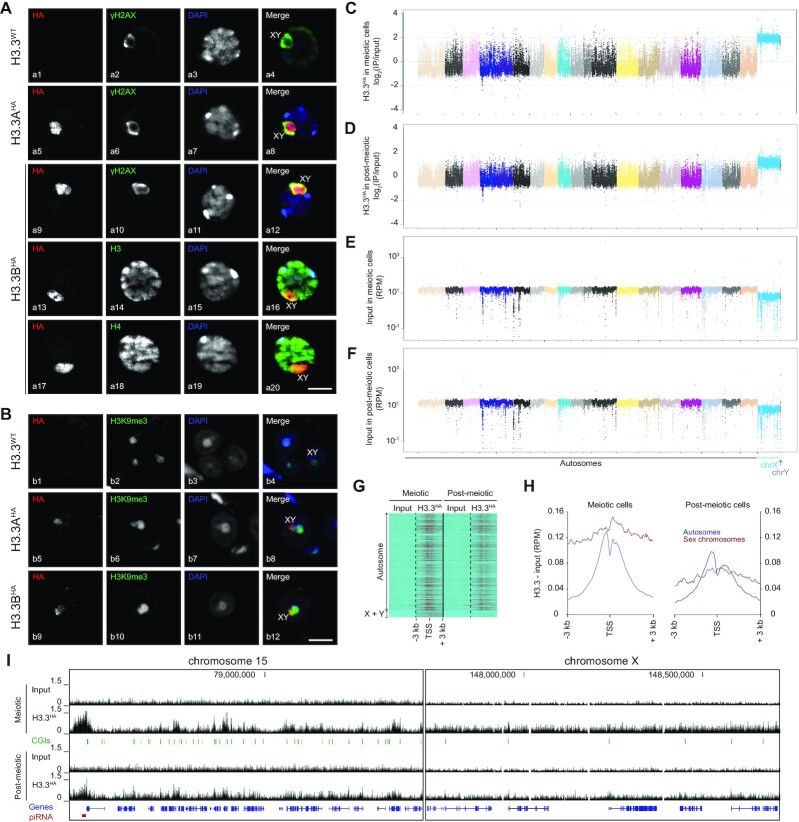
Enrichment and homogenous distribution of H3.3 along the sex chromosomes in spermatogenic cells. (A, B) Immunostaining of pachytene spermatocytes (**A**) and round spermatids (**B**) of H3.3^WT^ and epitope-tagged H3.3A^HA^ and H3.3B^HA^ mouse lines. The sex bodies are markedly enriched in H3.3A^HA^ (a5 and b5) and H3.3B^HA^ (a9, a13, a17 and b9) in pachytene spermatocytes and round spermatids of corresponding epitope-tagged H3.3 mouse lines. Nuclei were visualized with DAPI. Histone H3 and H4 were detected by anti-H3 and anti-H4 antibodies (a14 and a18). Anti-γH2AX (a2, a6 and a10) and anti-H3K9me3 (b2, b6 and b10) antibodies were used to visualize the sex body in pachytene spermatocytes and round spermatids, respectively. Scale bars, 8 μm (a20 and b12). (**C–F**) Manhattan plots (built using genome-wide ChIP data) showing the quantification of H3.3 associated with autosomes and sex chromosomes (C, D) and input samples (E, F) in meiotic (C, E) and post-meiotic cells (D, F). Note that only the sex chromosomes are found enriched with H3.3. (G, H) Heatmaps (**G**) and distribution pattern (**H**) of H3.3 relative to the TSS of autosomes and sex chromosomes in both meiotic and post-meiotic cells. (**I**) Genome browser view showing the distribution pattern and enrichment of H3.3 in chromosome 15 and X-chromosome in both meiotic and post-meiotic cells.

To shed further light on the implication of H3.3B in spermatogenesis, we next aimed to determine the genome-wide distribution pattern of H3.3. With this in mind, we carried out both immuno-fluorescence (IF) and native ChIP-seq experiments in epitope tagged FLAG-HA H3.3A and H3.3B mouse lines using commercially available anti-HA and anti-FLAG antibodies (Figure 5). For the ChIP-seq experiments, we purified by double immuno-affinity the native H3.3 mononucleosomes from micrococcal nuclease digested meiotic and post-meiotic nuclei (10). The IF data showed a clear enrichment of both epitope tagged H3.3A and H3.3B in the sex body (consisting of the X and Y chromosomes) of both pachytene spermatocytes and round spermatids (Figure 5A, B) as observed with the endogenous untagged proteins (Figure 3). This was further confirmed by the meiotic and post-meiotic cell ChIP-seq data, which showed, compared to autosomes, a four-fold enrichment of H3.3 in the sex chromosomes (Figure 5C, D). Interestingly, the percentage of detected reads for the sex chromosomes in the input fractions of both meiotic and post-meiotic cells was clearly smaller compared to those of the autosomes (Figure 5E, F). This would reflect lower accessibility of the sex chromosomes to the micrococcal nuclease and consequently, higher level of compaction of both of them. In the autosomes, H3.3 peaked at two sites around the TSS (Figure 5G). The picture is, however, clearly different in the sex chromosomes in both meiotic and post-meiotic cells. No enrichment of H3.3 is observed on promoters and gene bodies (Figure 5H). In fact, H3.3 showed a completely uniform distribution along the sex chromosome genes (Figure 5H, I). We attributed this to the transcriptional repressive status of the sex chromosomes in meiotic cells and in its minor role in the direct control of transcription.

The Manhattan plots, showing the transcription pattern for each individual chromosome, are presented in Supplementary Figure S3b-e. As expected, the meiotic/post-meiotic transition is associated with both up- and down-regulation of very large number of genes in each individual autosome (Supplementary Figure S3b). The absence of H3.3B does not affect the general up- and down-regulation pathway of the transcriptional autosome reprogramming (Supplementary Figure S3c). The meiotic/post-meiotic transition in WT cells is associated with massive up-regulation of the genes of the sex chromosomes in the post-meiotic cells (Supplementary Figure S3b), a result in agreement with the reported data (54). Again, H3.3 appeared not to affect the ‘directionality’ of this process (Supplementary Figure S3c). The comparison of the gene expression patterns in WT and H3.3B null both meiotic and post-meiotic cells revealed that all autosomes exhibited ‘two directional’ (up and down) alterations in their expression. However, the sex chromosomes showed distinct behavior, since essentially all their genes are found only up-regulated in the absence of H3.3B (Supplementary Figure S3d, e). We concluded that: (i) the association of H3.3B affects the sex chromosomes in a distinct manner compared to the autosomes and (ii) H3.3B behaves as a specific general repressor for the whole sex chromosomes.

### H3.3B and regulation of piRNA expression

**Figure 6.**
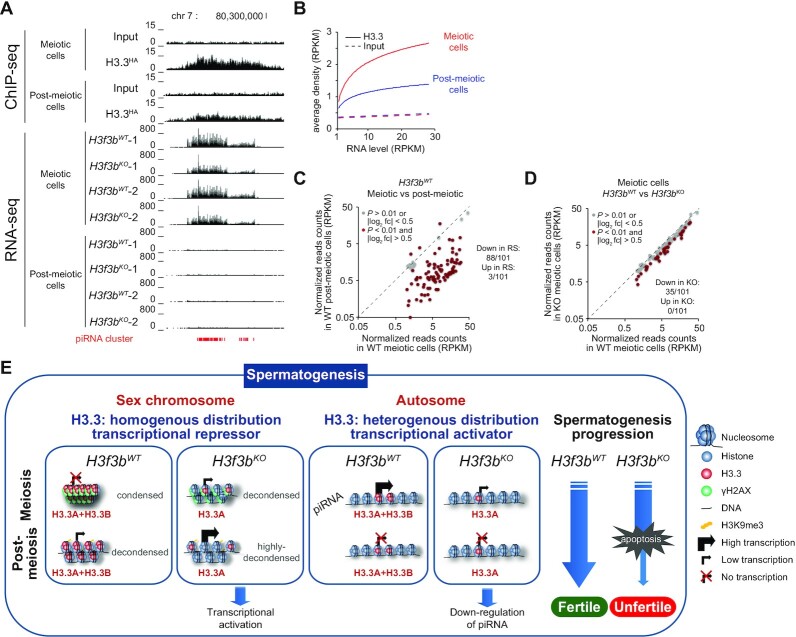
H3.3 regulates the expression of piRNA in meiotic cells. (**A**) Upper part. Double immunoaffinity purification of the HA-FLAG-tagged H3.3 mononucleosomes, followed by massive sequencing of ‘immunopurified’ nucleosomal DNA (ChIP-seq) were carried out to determine the genome-wide localization of H3.3. Example of strong H3.3 enrichment on piRNA cluster located on chromosome 7 in both meiotic and post-meiotic cells; lower part, expression profiles (RNA-seq) of the indicated piRNA cluster (indicated in red) from chromosome 7 in *H3f3b^WT^* and *H3f3b^KO^* meiotic and post-meiotic cells. In both cases two biologically independent experiments were presented. (**B**) The genome-wide expression level of piRNA clusters positively correlates with the amount of associated H3.3 in both meiotic and post-meiotic cells. (**C**) The meiotic/post-meiotic transition is associated with a ∼10-fold decrease in piRNA expression. (**D**) The absence of H3.3B is accompanied with a decrease of ∼25% of the expression of the piRNA clusters in meiotic cells. (**E**) Schematics depicting the dual role of H3.3 in spermatogenesis.

piRNA are playing an important role in spermatogenesis (24,26). To shed light if H3.3B could be involved in the regulation of their expression, we first analyzed in depth the distribution pattern of H3.3. Interestingly, the results unambiguously show that the piRNA clusters are enriched of H3.3 (Figure 6A, Supplementary Table S3, ChIP-seq panel). In addition, the H3.3 density followed piRNA cluster transcription (Figure 6B). The H3.3 enrichment on piRNA clusters is higher in meiotic cells compared to post-meiotic cells that would reflect the smaller level of H3.3 expression in the last ones (Figure 6A, RNA-seq panel and Figure 6B). The transition meiotic/post-meiotic cells is associated with massive repression of the piRNA cluster expression (88 of the 101 analyzed piRNA clusters were ∼10-fold down-regulated, Figure 6C) and their expression is hardly detected in post-meiotic cells.

Notably, the absence of H3.3B led to substantial transcriptional repression of the piRNA clusters in the meiotic cells (Figure 6A, Supplementary Table S3, RNA-seq panel). Quantification showed a ∼25% decrease in transcription level for one-third of the piRNA clusters in *H3f3b* null meiotic cells (35 of the 101 analyzed were significantly down-regulated, Figure 6D). In summary, the data showed that: (i) piRNA clusters were enriched of H3.3, (ii) higher is the H3.3 density, higher is the transcription of the piRNA clusters and (iii) the depletion of the H3.3B in meiotic cells led to down-regulation of a large part of the piRNA clusters. We concluded that H3.3B, probably through specific piRNA cluster chromatin organization, assists the efficient expression of the piRNA clusters.

### H3.3B is involved in the control of expression of specific LTR retrotransposons

The repression of the transposable element expression is crucial for spermatogenesis. With this in mind, we first analyzed genome-wide how H3.3B affects the transcription patterns of repetitive elements at the meiotic/post-meiotic transition. Of note, a massive down-regulation of repetitive elements expression is observed in post-meiotic cells compared to meiotic cells (Supplementary Figure S4a–f). Whereas the absence of H3.3B did not affect the general pattern of transposable element expression at the meiotic/post-meiotic transition (Supplementary Figure S4a–f), we found a specific and significant up-regulation of the RLTR10B and RLTR10B2 retrotranspons in both meiotic and post-meiotic cells (Supplementary Figure S4d–f). ChIP-seq analysis showed that these two retroelement families are enriched in H3.3 (Supplementary Figure SSupplementary Figure S4g). Taken as a whole these results suggest that H3.3B is implicated in the repression of both RLTR10B and RLTR10B2 retrotransposons.

## DISCUSSION

In this work we have carried out an in-depth study on the role of the histone variant H3.3 in spermatogenesis. The use of transgenic *H3f3a* and *H3f3b* conditional knock-out/knock-in (cKO/KI) mouse lines combined with a large number of genome-wide approaches has allowed to demonstrate that H3.3B, but not H3.3A, is a key factor in the spermatogenic process.

The *H3f3b* null mice exhibited very severe infertility, while the *H3f3a* null mice did not show any fertility problem and behaved as the WT mice. The seminiferous tubules of the *H3f3b* null males contained smaller number of spermatozoa. The morphology of their spermatozoa was highly altered and their sperm concentration in the epididymis was strongly decreased. Notably, the meiotic/post-meiotic cells ratio was largely perturbed and the production of post-meiotic cells was heavily reduced in *H3f3b* null males.

Meiotic sex-chromosome inactivation (MSCI) correlates with the presence of the phosphorylated form of the histone variant H2A.X (γH2AX) in sex chromosome chromatin (Figure 3A) (29). H2AX is phosphorylated by the kinase ATR, which recruitment depends on BRCA1 (46). After MSCI induction, both removal of conventional H3 and accumulation of H3.3 in the sex body were observed (55). This led to the hypothesis that both γH2AX and H3.3 might be involved in sex body formation, sex chromosome condensation and their silencing (29,55). It is worthwhile to note, however, that these data have correlative character only and no formal demonstration that both H3.3 and γH2AX are required for MSCI was reported to date. For example, γH2AX might only mark the induction of MSCI and not to be directly involved in the process of sex chromosome condensation. Indeed, *Xenopus* sperm decompaction (a process opposite of chromosome condensation) in *Xenopus* egg extracts correlates with the recruitment of γH2AX and γH2AX is present in both actively dividing, unperturbed embryos in the absence and presence of DNA damage (56,57). The claims on the involvement of H3.3 in the X-chromosome inactivation could be similarly criticized, since there are no reported data on the casual relationship between the accumulation of H3.3 on the sex body and the repression of the transcription of the sex chromosomes in meiosis.

Herein, we used a battery of genome wide methods to analyze in depth the role of H3.3 in spermatogenesis. Transcriptome studies revealed very limited clustering of the affected genes by the absence of H3.3B in both meiotic and post-meiotic cells, suggesting a minor implication of H3.3B in the control of specific genes at the genome-wide scale. Our immunofluorescence and ChIP-seq experiments showed a very strong (exceeding four-fold in meiotic cells) enrichment of the endogenous H3.3 on the whole sex body, a result in agreement with the past report where transgenic mouse models, expressing exogenous H3.3, were used (55). We demonstrated that H3.3 is localized uniformly along the sex chromosomes, in contrast to the autosomes, where sites of H3.3 enrichment were observed, in particular at active promoters and enhancers. More importantly, Manhattan plot analysis revealed that the absence of H3.3B is associated with selective up-regulation of the expression of the whole sex chromosomes. This study therefore demonstrates unequivocally for the first time specific transcriptional repression induced by H3.3 in the sex chromosomes during spermatogenesis. Since both sex chromosomes are, compared to the autosomes, less accessible to micrococcal nucleases, this could reflect an H3.3-mediated higher compaction of both X and Y chromosomes associated with the uniform distribution pattern of H3.3 along their length. Xist, a long non-coding RNA, is coating the X-chromosome along its entire length and is a major effector in its inactivation during mammalian development (58). Our data show that H3.3 is playing similar to Xist role, but during spermatogenesis. Therefore, nature has chosen two fundamental different effectors to inactivate the X chromosome: a long RNA (Xist) and the histone variant H3.3 during mammalian embryogenesis and spermatogenesis, respectively. Of note, alterations of sex chromosomes expression were correlated with male infertility and sperm head anomalies (50,59) which is in full agreement with our data showing male infertility and strongly altered morphology of the spermatozoa of the *H3f3b* null mice.

We have also analyzed the expression of the repetitive elements during spermatogenesis. The meiotic/post-meiotic transition was associated with massive repression of the repetitive elements. H3.3 was found enriched only on two families of repetitive elements, the RLTR10B and RLTR10B2 retrotransposons, that are likely implicated in infertility (25,60). Depletion of H3.3B led to up-regulation of both retrotransposons in both meiotic and post-meiotic cells. This suggested that H3.3B, as in the case of sex chromosomes, is playing a repressive role in the regulation of expression of RLTR10B and RLTR10B2 retrotransposons.

Our ChIP-seq data clearly demonstrated that the piRNA clusters are highly enriched of H3.3. The H3.3 enrichment followed the expression level of the clusters. Depletion of H3.3B led, in contrast to the case of sex chromosomes and RLTR10B and RLTR10B2 retrotransposons, to large diminution of the expression of piRNA, suggesting that the presence of H3.3B assisted piRNA expression. The observed phenotype of infertility of *H3f3b^KO^* male is therefore due to ‘convergent’ defects: upregulation of genes located in sex chromosome, upregulation of RLTR10B-RLTR10B2 retrotransposons and down regulation of piRNA pathway. It is worth noting that any of these defects, when occurs alone, leads to infertility (50,59,60).

In summary, our data show a specific implication of H3.3B in spermatogenesis. Since both H3.3A and H3.3B have identical amino-acid sequence, we speculated that this implication of H3.3B reflects the higher amount of this protein compared to H3.3A during spermatogenesis. Indeed, H3.3B represents the majority of the total H3.3 at the RNA and protein level as evidenced by immunofluorescence, immunohistochemistry, RNA-seq and western blotting analyses (Supplementary Figure S5a–d). In addition, depletion of H3.3B, did not affect the expression level of H3.3A (Supplementary Figure S5c, d), further supporting our hypothesis.

In addition, our data reveal that H3.3B could play either a repressive role or could assist expression (Figure 6E) in the same cell types. The repressive role of H3.3B is exemplified in the case of the sex chromosomes and the RLTR10B and RLTR10B2 retrotransposon families. However, H3.3B assists the expression of the piRNA at genome-wide level. This very interesting phenomenon illustrates the ‘Janus’ (double face) role of H3.3. This suggests that H3.3B might have intrinsic ability to affect in a distinct way chromatin structure depending on its distribution pattern, abundance, post-translational modifications and assembly of specific local nucleosome organization (Figure 6E). Alternatively, H3.3B could be viewed simply as a ‘replacement’ histone, that the spermatogenic cell uses to replace the conventional H3 at specific already distinctly organized chromatin structures. This role could be fulfilled since H3.3, in contrast to H3, is expressed at any phase at the cell cycle and the cell could efficiently use such ‘replacement piece’ at any time. It is important to note that the function of H3.3 is independent of DNA methylation. No change in DNA methylation or hydroxymethylation profiles could be detected by DIP-seq at neither misregulated genes (Supplementary Figure S6), DNA repeats, piRNA clusters nor sex chromosomes (Supplementary Figure S7). Additional properties of H3.3 relate to the proportion of PTMs associated with active or repressive chromatin. Indeed, H3.3K4me3 mark is associated with transcription activation while H3.3K9me3 is associated with repression by the co-repressor complex KAP1 (61). Furthermore, the requirement of H3.3 histone post-translational modifications during gametogenesis warrant further investigations.

## DATA AVAILABILITY

The ChIP-seq, DIP-seq and RNA-seq datasets have been deposited in the Gene Expression Omnibus (GEO; http://www.ncbi.nlm.nih.gov/geo/) under the accession number GSE116373.

## Supplementary Material

gkac541_Supplemental_Files

## Notes

Present address: Bertrand Favier, Univ. Grenoble Alpes, CNRS, UMR 5525, VetAgro Sup, Grenoble INP, TIMC, 38000 Grenoble, France.

## References

[1] Luger K. , Mäder A.W., Richmond R.K., Sargent D.F., Richmond T.J. Crystal structure of the nucleosome core particle at 2.8 a resolution. Nature. 1997; 389:251–260. 9305837 10.1038/38444

[2] Boulard M. , Bouvet P., Kundu T.K., Dimitrov S. Histone variant nucleosomes: structure, function and implication in disease. Subcell. Biochem. 2007; 41:71–89. 17484124

[3] Talbert P.B. , Henikoff S. Histone variants–ancient wrap artists of the epigenome. Nat. Rev. Mol. Cell Biol. 2010; 11:264–275. 20197778 10.1038/nrm2861

[4] van Holde K. Chromatin. 1988; Berlin, Germany Springer-Verlag KG.

[5] Latrick C.M. , Marek M., Ouararhni K., Papin C., Stoll I., Ignatyeva M., Obri A., Ennifar E., Dimitrov S., Romier C. et al. Molecular basis and specificity of H2A.Z-H2B recognition and deposition by the histone chaperone YL1. Nat. Struct. Mol. Biol. 2016; 23:309–316. 26974126 10.1038/nsmb.3189

[6] Obri A. , Ouararhni K., Papin C., Diebold M.L., Padmanabhan K., Marek M., Stoll I., Roy L., Reilly P.T., Mak T.W. et al. ANP32E is a histone chaperone that removes H2A.Z from chromatin. Nature. 2014; 505:648–653. 24463511 10.1038/nature12922

[7] Shuaib M. , Ouararhni K., Dimitrov S., Hamiche A. HJURP binds CENP-A via a highly conserved N-terminal domain and mediates its deposition at centromeres. Proc. Natl. Acad. Sci. U.S.A. 2010; 107:1349–1354. 20080577 10.1073/pnas.0913709107 PMC2824361

[8] Dunleavy E.M. , Almouzni G., Karpen G.H. H3.3 is deposited at centromeres in s phase as a placeholder for newly assembled CENP-A in G(1) phase. Nucleus. 2011; 2:146–157. 21738837 10.4161/nucl.2.2.15211 PMC3127096

[9] Goldberg A.D. , Banaszynski L.A., Noh K.M., Lewis P.W., Elsaesser S.J., Stadler S., Dewell S., Law M., Guo X., Li X. et al. Distinct factors control histone variant H3.3 localization at specific genomic regions. Cell. 2010; 140:678–691. 20211137 10.1016/j.cell.2010.01.003 PMC2885838

[10] Drane P. , Ouararhni K., Depaux A., Shuaib M., Hamiche A. The death-associated protein DAXX is a novel histone chaperone involved in the replication-independent deposition of h3.3. Genes Dev. 2010; 24:1253–1265. 20504901 10.1101/gad.566910 PMC2885661

[11] Krimer D.B. , Cheng G., Skoultchi A.I. Induction of H3.3 replacement histone mRNAs during the precommitment period of murine erythroleukemia cell differentiation. Nucleic Acids Res. 1993; 21:2873–2879. 8332496 10.1093/nar/21.12.2873 PMC309673

[12] Szenker E. , Ray-Gallet D., Almouzni G. The double face of the histone variant H3.3. Cell Res. 2011; 21:421–434. 21263457 10.1038/cr.2011.14 PMC3193428

[13] Ricketts M.D. , Frederick B., Hoff H., Tang Y., Schultz D.C., Singh Rai T., Grazia Vizioli M., Adams P.D., Marmorstein R Ubinuclein-1 confers histone H3.3-specific-binding by the HIRA histone chaperone complex. Nat. Commun. 2015; 6:7711. 26159857 10.1038/ncomms8711 PMC4510971

[14] Jin C. , Felsenfeld G. Nucleosome stability mediated by histone variants H3.3 and H2A.Z. Genes Dev. 2007; 21:1519–1529. 17575053 10.1101/gad.1547707 PMC1891429

[15] Ors A. , Papin C., Favier B., Roulland Y., Dalkara D., Ozturk M., Hamiche A., Dimitrov S., Padmanabhan K. Histone H3.3 regulates mitotic progression in mouse embryonic fibroblasts. Biochem. Cell. Biol. 2017; 95:491–499. 28177753 10.1139/bcb-2016-0190

[16] Jang C.W. , Shibata Y., Starmer J., Yee D., Magnuson T. Histone H3.3 maintains genome integrity during mammalian development. Genes Dev. 2015; 29:1377–1392. 26159997 10.1101/gad.264150.115 PMC4511213

[17] Huang C. , Zhu B. H3.3 turnover: a mechanism to poise chromatin for transcription, or a response to open chromatin?. Bioessays. 2014; 36:579–584. 24700556 10.1002/bies.201400005

[18] Sakai A. , Schwartz B.E., Goldstein S., Ahmad K. Transcriptional and developmental functions of the H3.3 histone variant in drosophila. Curr. Biol. 2009; 19:1816–1820. 19781938 10.1016/j.cub.2009.09.021 PMC2783816

[19] Yuen B.T. , Bush K.M., Barrilleaux B.L., Cotterman R., Knoepfler P.S. Histone H3.3 regulates dynamic chromatin states during spermatogenesis. Development. 2014; 141:3483–3494. 25142466 10.1242/dev.106450 PMC4197731

[20] Tang M.C. , Jacobs S.A., Mattiske D.M., Soh Y.M., Graham A.N., Tran A., Lim S.L., Hudson D.F., Kalitsis P., O’Bryan M.K. et al. Contribution of the two genes encoding histone variant h3.3 to viability and fertility in mice. PLoS Genet. 2015; 11:e1004964. 25675407 10.1371/journal.pgen.1004964 PMC4335506

[21] Couldrey C. , Carlton M.B., Nolan P.M., Colledge W.H., Evans M.J. A retroviral gene trap insertion into the histone 3.3A gene causes partial neonatal lethality, stunted growth, neuromuscular deficits and male sub-fertility in transgenic mice. Hum. Mol. Genet. 1999; 8:2489–2495. 10556297 10.1093/hmg/8.13.2489

[22] de Koning A.P. , Gu W., Castoe T.A., Batzer M.A., Pollock D.D. Repetitive elements may comprise over two-thirds of the human genome. PLoS Genet. 2011; 7:e1002384. 22144907 10.1371/journal.pgen.1002384 PMC3228813

[23] Papin C. , Ibrahim A., Gras S.L., Velt A., Stoll I., Jost B., Menoni H., Bronner C., Dimitrov S., Hamiche A. Combinatorial DNA methylation codes at repetitive elements. Genome Res. 2017; 27:934–946. 28348165 10.1101/gr.213983.116 PMC5453327

[24] Slotkin R.K. , Martienssen R. Transposable elements and the epigenetic regulation of the genome. Nat. Rev. Genet. 2007; 8:272–285. 17363976 10.1038/nrg2072

[25] Isbel L. , Srivastava R., Oey H., Spurling A., Daxinger L., Puthalakath H., Whitelaw E. Trim33 binds and silences a class of young endogenous retroviruses in the mouse testis; a novel component of the arms race between retrotransposons and the host genome. PLoS Genet. 2015; 11:e1005693. 26624618 10.1371/journal.pgen.1005693 PMC4666613

[26] Chuma S. , Nakano T. piRNA and spermatogenesis in mice. Philos. Trans. R. Soc. Lond. B Biol. Sci. 2013; 368:20110338. 23166399 10.1098/rstb.2011.0338 PMC3539364

[27] Meistrich M.L. , Hess R.A. Assessment of spermatogenesis through staging of seminiferous tubules. Methods Mol. Biol. 2013; 927:299–307. 22992924 10.1007/978-1-62703-038-0_27

[28] Turner J.M. , Mahadevaiah S.K., Fernandez-Capetillo O., Nussenzweig A., Xu X., Deng C.X., Burgoyne P.S. Silencing of unsynapsed meiotic chromosomes in the mouse. Nat. Genet. 2005; 37:41–47. 15580272 10.1038/ng1484

[29] Mahadevaiah S.K. , Turner J.M., Baudat F., Rogakou E.P., de Boer P., Blanco-Rodriguez J., Jasin M., Keeney S., Bonner W.M., Burgoyne P.S. Recombinational DNA double-strand breaks in mice precede synapsis. Nat. Genet. 2001; 27:271–276. 11242108 10.1038/85830

[30] Kanatsu-Shinohara M. , Toyokuni S., Shinohara T. Transgenic mice produced by retroviral transduction of male germ line stem cells in vivo. Biol. Reprod. 2004; 71:1202–1207. 15189822 10.1095/biolreprod.104.031294

[31] Hess R.A. , de Franca L.R. Spermatogenesis and cycle of the seminiferous epithelium. Adv. Exp. Med. Biol. 2008; 636:1–15. 19856159 10.1007/978-0-387-09597-4_1

[32] Bellve A.R. Purification, culture, and fractionation of spermatogenic cells. Methods Enzymol. 1993; 225:84–113. 8231890 10.1016/0076-6879(93)25009-q

[33] Buchou T. , Tan M., Barral S., Vitte A.L., Rousseaux S., Arechaga J., Khochbin S. Purification and analysis of male germ cells from adult mouse testis. Methods Mol. Biol. 2017; 1510:159–168. 27761820 10.1007/978-1-4939-6527-4_12

[34] Goutte-Gattat D. , Shuaib M., Ouararhni K., Gautier T., Skoufias D.A., Hamiche A., Dimitrov S. Phosphorylation of the CENP-A amino-terminus in mitotic centromeric chromatin is required for kinetochore function. Proc. Natl. Acad. Sci. U.S.A. 2013; 110:8579–8584. 23657009 10.1073/pnas.1302955110 PMC3666736

[35] Roulland Y. , Ouararhni K., Naidenov M., Ramos L., Shuaib M., Syed S.H., Lone I.N., Boopathi R., Fontaine E., Papai G. et al. The flexible ends of CENP-A nucleosome are required for mitotic fidelity. Mol. Cell. 2016; 63:674–685. 27499292 10.1016/j.molcel.2016.06.023

[36] Langmead B. , Trapnell C., Pop M., Salzberg S.L. Ultrafast and memory-efficient alignment of short DNA sequences to the human genome. Genome Biol. 2009; 10:R25. 19261174 10.1186/gb-2009-10-3-r25 PMC2690996

[37] Ibrahim A. , Papin C., Mohideen-Abdul K., Le Gras S., Stoll I., Bronner C., Dimitrov S., Klaholz B.P., Hamiche A. MeCP2 is a microsatellite binding protein that protects CA repeats from nucleosome invasion. Science. 2021; 372:eabd5581. 34324427 10.1126/science.abd5581

[38] Ye T. , Krebs A.R., Choukrallah M.A., Keime C., Plewniak F., Davidson I., Tora L. seqMINER: an integrated chip-seq data interpretation platform. Nucleic. Acids. Res. 2011; 39:e35. 21177645 10.1093/nar/gkq1287 PMC3064796

[39] Trapnell C. , Pachter L., Salzberg S.L. TopHat: discovering splice junctions with RNA-Seq. Bioinformatics. 2009; 25:1105–1111. 19289445 10.1093/bioinformatics/btp120 PMC2672628

[40] Anders S. , Huber W. Differential expression analysis for sequence count data. Genome Biol. 2010; 11:R106. 20979621 10.1186/gb-2010-11-10-r106 PMC3218662

[41] Love M.I. , Huber W., Anders S. Moderated estimation of fold change and dispersion for RNA-seq data with DESeq2. Genome Biol. 2014; 15:550. 25516281 10.1186/s13059-014-0550-8 PMC4302049

[42] Hochberg Y. , Benjamini Y. More powerful procedures for multiple significance testing. Stat. Med. 1990; 9:811–818. 2218183 10.1002/sim.4780090710

[43] Barral S. , Morozumi Y., Tanaka H., Montellier E., Govin J., de Dieuleveult M., Charbonnier G., Coute Y., Puthier D., Buchou T. et al. Histone variant H2A.L.2 guides transition protein-dependent protamine assembly in male germ cells. Mol. Cell. 2017; 66:89–101. 28366643 10.1016/j.molcel.2017.02.025

[44] Govin J. , Escoffier E., Rousseaux S., Kuhn L., Ferro M., Thevenon J., Catena R., Davidson I., Garin J., Khochbin S. et al. Pericentric heterochromatin reprogramming by new histone variants during mouse spermiogenesis. J. Cell Biol. 2007; 176:283–294. 17261847 10.1083/jcb.200604141 PMC2063955

[45] Tarsounas M. , Morita T., Pearlman R.E., Moens P.B. RAD51 and DMC1 form mixed complexes associated with mouse meiotic chromosome cores and synaptonemal complexes. J. Cell Biol. 1999; 147:207–220. 10525529 10.1083/jcb.147.2.207 PMC2174216

[46] Turner J.M. , Aprelikova O., Xu X., Wang R., Kim S., Chandramouli G.V., Barrett J.C., Burgoyne P.S., Deng C.X. BRCA1, histone H2AX phosphorylation, and male meiotic sex chromosome inactivation. Curr. Biol. 2004; 14:2135–2142. 15589157 10.1016/j.cub.2004.11.032

[47] Bramlage B. , Kosciessa U., Doenecke D Differential expression of the murine histone genes H3.3A and H3.3B. Differentiation. 1997; 62:13–20. 9373943 10.1046/j.1432-0436.1997.6210013.x

[48] Eijpe M. , Offenberg H., Jessberger R., Revenkova E., Heyting C. Meiotic cohesin REC8 marks the axial elements of rat synaptonemal complexes before cohesins SMC1beta and SMC3. J. Cell Biol. 2003; 160:657–670. 12615909 10.1083/jcb.200212080 PMC2173354

[49] Bisig C.G. , Guiraldelli M.F., Kouznetsova A., Scherthan H., Hoog C., Dawson D.S., Pezza R.J. Synaptonemal complex components persist at centromeres and are required for homologous centromere pairing in mouse spermatocytes. PLoS Genet. 2012; 8:e1002701. 22761579 10.1371/journal.pgen.1002701 PMC3386160

[50] Ellis P.J. , Clemente E.J., Ball P., Toure A., Ferguson L., Turner J.M., Loveland K.L., Affara N.A., Burgoyne P.S. Deletions on mouse yq lead to upregulation of multiple X- and Y-linked transcripts in spermatids. Hum. Mol. Genet. 2005; 14:2705–2715. 16087683 10.1093/hmg/ddi304

[51] Baarends W.M. , Hoogerbrugge J.W., Roest H.P., Ooms M., Vreeburg J., Hoeijmakers J.H., Grootegoed J.A. Histone ubiquitination and chromatin remodeling in mouse spermatogenesis. Dev. Biol. 1999; 207:322–333. 10068466 10.1006/dbio.1998.9155

[52] Khalil A.M. , Boyar F.Z., Driscoll D.J. Dynamic histone modifications mark sex chromosome inactivation and reactivation during mammalian spermatogenesis. Proc. Natl. Acad. Sci. U.S.A. 2004; 101:16583–16587. 15536132 10.1073/pnas.0406325101 PMC534513

[53] Motzkus D. , Singh P.B., Hoyer-Fender S. M31, a murine homolog of drosophila HP1, is concentrated in the XY body during spermatogenesis. Cytogenet. Cell Genet. 1999; 86:83–88. 10516442 10.1159/000015418

[54] Moretti C. , Vaiman D., Tores F., Cocquet J. Expression and epigenomic landscape of the sex chromosomes in mouse post-meiotic male germ cells. Epigenetics Chromatin. 2016; 9:47. 27795737 10.1186/s13072-016-0099-8 PMC5081929

[55] van der Heijden G.W. , Derijck A.A., Posfai E., Giele M., Pelczar P., Ramos L., Wansink D.G., van der Vlag J., Peters A.H., de Boer P. Chromosome-wide nucleosome replacement and H3.3 incorporation during mammalian meiotic sex chromosome inactivation. Nat. Genet. 2007; 39:251–258. 17237782 10.1038/ng1949

[56] Dimitrov S. , Dasso M.C., Wolffe A.P. Remodeling sperm chromatin in xenopus laevis egg extracts: the role of core histone phosphorylation and linker histone B4 in chromatin assembly. J. Cell Biol. 1994; 126:591–601. 8045925 10.1083/jcb.126.3.591 PMC2120139

[57] Shechter D. , Chitta R.K., Xiao A., Shabanowitz J., Hunt D.F., Allis C.D. A distinct H2A.X isoform is enriched in xenopus laevis eggs and early embryos and is phosphorylated in the absence of a checkpoint. Proc. Nat. Acad. Sci. U.S.A. 2009; 106:749–754. 10.1073/pnas.0812207106 PMC2630098 19131518

[58] da Rocha S.T. , Heard E. Novel players in x inactivation: insights into Xist-mediated gene silencing and chromosome conformation. Nat. Struct. Mol. Biol. 2017; 24:197–204. 28257137 10.1038/nsmb.3370

[59] Reynard L.N. , Turner J.M. Increased sex chromosome expression and epigenetic abnormalities in spermatids from male mice with y chromosome deletions. J. Cell Sci. 2009; 122:4239–4248. 19861498 10.1242/jcs.049916 PMC2776507

[60] Tarabay Y. , Achour M., Teletin M., Ye T., Teissandier A., Mark M., Bourc’his D., Viville S. Tex19 paralogs are new members of the piRNA pathway controlling retrotransposon suppression. J. Cell Sci. 2017; 130:1463–1474. 28254886 10.1242/jcs.188763

[61] Rowe H.M. , Kapopoulou A., Corsinotti A., Fasching L., Macfarlan T.S., Tarabay Y., Viville S., Jakobsson J., Pfaff S.L., Trono D TRIM28 repression of retrotransposon-based enhancers is necessary to preserve transcriptional dynamics in embryonic stem cells. Genome Res. 2013; 23:452–461. 23233547 10.1101/gr.147678.112 PMC3589534

