# Supplementary material for: Dual role of histone variant H3.3B in spermatogenesis: positive regulation of piRNA transcription and implication in X-chromosome inactivation
Source: Nucleic Acids Res. 2022 Jun 29;50(13):7350–66. doi: 10.1093/nar/gkac541 (PMC9303386; doi:10.1093/nar/gkac541)
Supplement: gkac541_Supplemental_Files [file gkac541_supplemental_files.zip › Supplementary Data.pdf]

**Supplementary Data_Fontaine et al.**

**Supplementary Figure legends**

**Supplementary Figure 1.** (**a-b**) Generation of *H3f3a*$^{KO}$ (***a***) and *H3f3b*$^{KO}$ mouse models (***b***). Upper panels, wild-type *H3f3a and H3f3b* genes structure. The open reading frames are indicated by black boxes. Middle panels, a DNA element encoding the FLAG–FLAG–HA amino acids was inserted in frame with the N-terminus of H3.3A or H3.3B (H3.3A$^{HA}$ and H3.3B$^{HA}$). In addition, two loxP sites were inserted (red arrowheads) to flank exon 2 (**a**) or all coding exons (**b**) of the *H3f3a* and *H3f3b* genes, respectively. Lower panels, structure of the loss of function *H3f3a* knock-out (*H3f3a*$^{KO}$) and *H3f3b* knock-out (*H3f3b*$^{KO}$) alleles, after Cre recombinase expression. (**c-f**) Genotyping of the indicated mouse lines. The length of the PCR products amplified with the primers shown in (**a**) and (**b**) are indicated. (**g**) *H3f3b* null males exhibit slightly reduced sperm vitality, (**h**) increased DNA fragmentation, (**i**) strongly altered morphology, (**j,k**) including flagella anomalies, (**l,m**) head anomalies, (**n**) strong DNA decompaction (increasing from 1% to 10%), and (**o**) smaller amount of associated protamine. Two biological replicates were used for sperm analyses. (**p-r**) RT-qPCR analyses of *Tnp1* (**p**), *Prm1* (**q**) and *Prm2* (**r**) mRNA levels in round spermatids of *H3f3b*$^{WT}$ and H3f3b$^{KO}$ males. RNA was extracted from 3 biological replicates. Since the expression of transition protein TNP1 and both protamine 1 and protamine 2 were not altered, the smaller amount of incorporated protamines could reflect the altered ability of protamines to associate with sperm DNA in the absence of H3.3B. (**g-i, k, m-r**) *P*-values were calculated from Student's unpaired *t*-tests.

**Supplementary Figure 2.** (**a-b**) The absence of H3.3B results in a decreased number of post-meiotic cells. Acrosome visualization by PNA staining (red) in WT, *H3f3a* and *H3f3b* null testis sections at stage II (**a**) and stage IX (**b**). DNA is stained with DAPI. **a7-a9** and **b7-b9** correspond to the enlargement of the white square in **a1-a3** and **b1-b3**, respectively. Scale bars, 70 µm (**a6, b6**) and 30 µm (**b6, b9**). (**c-d**) Loss of *H3f3b* did not affect the number of spermatogonia in the seminiferous tubules. (**c**) WT and *H3f3b*$^{KO}$ testis sections stained with DAPI (blue) for nucleus detection and with anti-PLZF (green) for spermatogonia visualization. Scale bars: 120 µm (**b3**), 30 µm (**b4**). (**d**) PLZF positive cells counting were carried out in seminiferous tubules cross sections (n=100) of WT and *H3f3b*$^{KO}$ testis. *P*-values were calculated from Student's unpaired *t*-tests.

**Supplementary Figure 3**. (**a**) RT-qPCR analyses of gene expression levels in *H3f3b*$^{WT}$ and *H3f3b*$^{KO}$ meiotic and post-meiotic cells. RNA was extracted from 3 biological replicates. *Hand2* and *Znf811* genes are up-regulated, and *Abcg8*, *Fmnl1* and *Ptpro* genes are down-regulated in the absence of H3.3b, as detected by RNA-seq analyses. (**b-e**) The absence of H3.3 is associated with increased gene expression level of the entire sex chromosomes. Manhattan plots (built on massive RNA-seq data) showing the changes in the expression level of individual chromosomes in the meiotic/post-meiotic transition in the presence (**b**) or absence (**c**) of H3.3B. The **d** and **e** graphs reveal the alterations in the expression pattern of individual chromosomes in the absence of H3.3B in meiotic and post-meiotic cells, respectively. Note that all chromosomes, except the sex ones, show both up-regulation and down-regulation of expression in the absence of H3.3B. The genome wide expressions of the sex chromosomes are only up-regulated.

**Supplementary Figure 4**. Genome-wide transcriptome analysis of the repetitive elements in meiotic and post-meiotic cells in the absence of H3.3B. (**a,b**) Scatter plots comparing repetitive elements expression profiles of meiotic and post-meiotic cells in the presence (**a**) or absence (**b**) of H3.3B. Red dots indicate differentially expressed repetitive elements. (**c**) Examples of silencing of retroelements in post-meiotic cells relative to meiotic cells in $H3f3b^{WT}$ and $H3f3b^{KO}$ males. The family of the repetitive elements are indicated. $^{**}P < 10^{-60}$. (**d,e**) Scatter plots comparing repetitive elements expression profiles of $H3f3b^{WT}$ versus $H3f3b^{KO}$ in meiotic (**d**) and post-meiotic cells (**e**). Red dots indicate differentially expressed repetitive elements. (**f**) The absence of H3.3B is associated with an increased expression level of both RLTR10B and RLTR10B2 retrotransposon families in both meiotic and post-meiotic cells. $^{*}P < 10^{-10}$. (**g**) RLTR10B and RLTR10B2 repetitive elements are enriched in H3.3 in both meiotic and post-meiotic cells.

**Supplementary Figure 5.** H3.3 is mainly expressed from *H3f3b* gene in spermatocytes and spermatids. (**a**) H3.3 staining in pachytene spermatocytes and round spermatids of $H3f3b^{WT}$ and $H3f3b^{KO}$ mice. Anti-SCP3 antibody was used for the identification of pachytene spermatocytes. The round spermatids were identified by the very compacted chromatin visualized by DAPI staining. Scale bar, 8 µm. (**b**) Assessment of H3.3 quantity by immunochemistry performed on testis sections from $H3f3b^{WT}$, $H3f3a^{KO}$ and $H3f3b^{KO}$ mice. W. hile similar H3.3 staining (labeling in brown) were observed in $H3f3b^{WT}$ and $H3f3a^{KO}$ testis, the H3.3 staining was greatly reduced in $H3f3b^{KO}$ testis. Scale bar, 200 µm (**c**) Bar graph representing the cumulative RNA level of *H3.1*, *H3.2* and *H3.3* genes in meiosis and post-meiosis, according to RNA-seq data. (**d**) Western blotting analysis of H3.3 in meiotic and post-meiotic cells WT or KO for *H3.3b.* For quantification, protein amount was normalized to total protein loading by UV activation and visualization.

**Supplementary Figure 6.** Average 5mC and 5hmC signals within gene bodies of stably, up-regulated or down-regulated genes in the absence of H3.3B in meiosis and post-meiosis cells WT and KO for *H3.3b*. Tag densities were collected in 100-bp sliding windows spanning 2 kb (divided in 10 bins) of the length-normalized gene bodies (divided in 40 bins).

**Supplementary Figure 7.** (**a**) Scatter plots comparing cytosine modifications enrichment at DNA repeats between $H3f3b^{WT}$ and $H3f3b^{KO}$ cells. (**b**) Average 5mC and 5hmC signals along piRNA clusters in meiotic and post-meiotic cells WT or KO for *H3.3b*. Tag densities were collected in 500-bp sliding windows spanning 20 kb (divided in 20 bins) of the length-normalized piRNA clusters (divided in 20 bins). (**c**) Manhattan plots (built using DIP-seq data) showing the log2-ratio between WT and KO cells in 5mC and 5hmC enrichment along the chromosomes in meiosis and post-meiosis.

**Supplementary Table 1**. Listing of the most significantly mis-regulated genes (|log2-ratio KO/WT| > 1, $P < 0.01$) in $H3f3b^{KO}$ meiotic cells. The following information are indicated for each gene: Ensemble gene id, gene name, log2 fold change, p-value adjusted for multi-testing.

**Supplementary Table 2**. Listing of the most significantly mis-regulated (|log2-ratio KO/WT| > 1, $P < 0.01$) in $H3f3b^{KO}$ post-meiotic cells. The following information are indicated for each gene: Ensemble gene id, gene name, log2 fold, p-value adjusted for multi-testing.

**Supplementary Table 3**. Listing of the most mis-regulated piRNA in the meiotic/ post-meiotic transition.

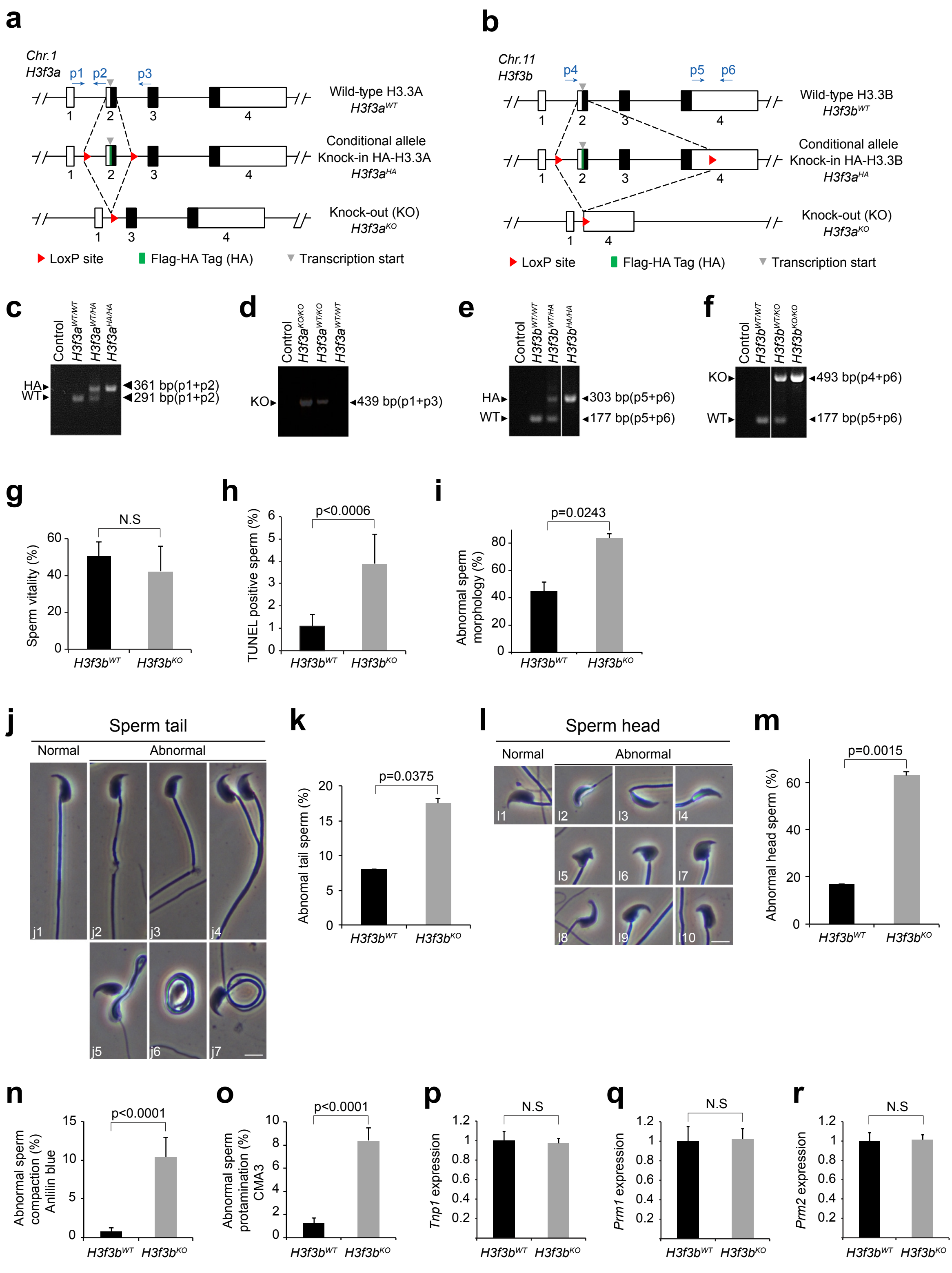


# Supplementary Figure 1

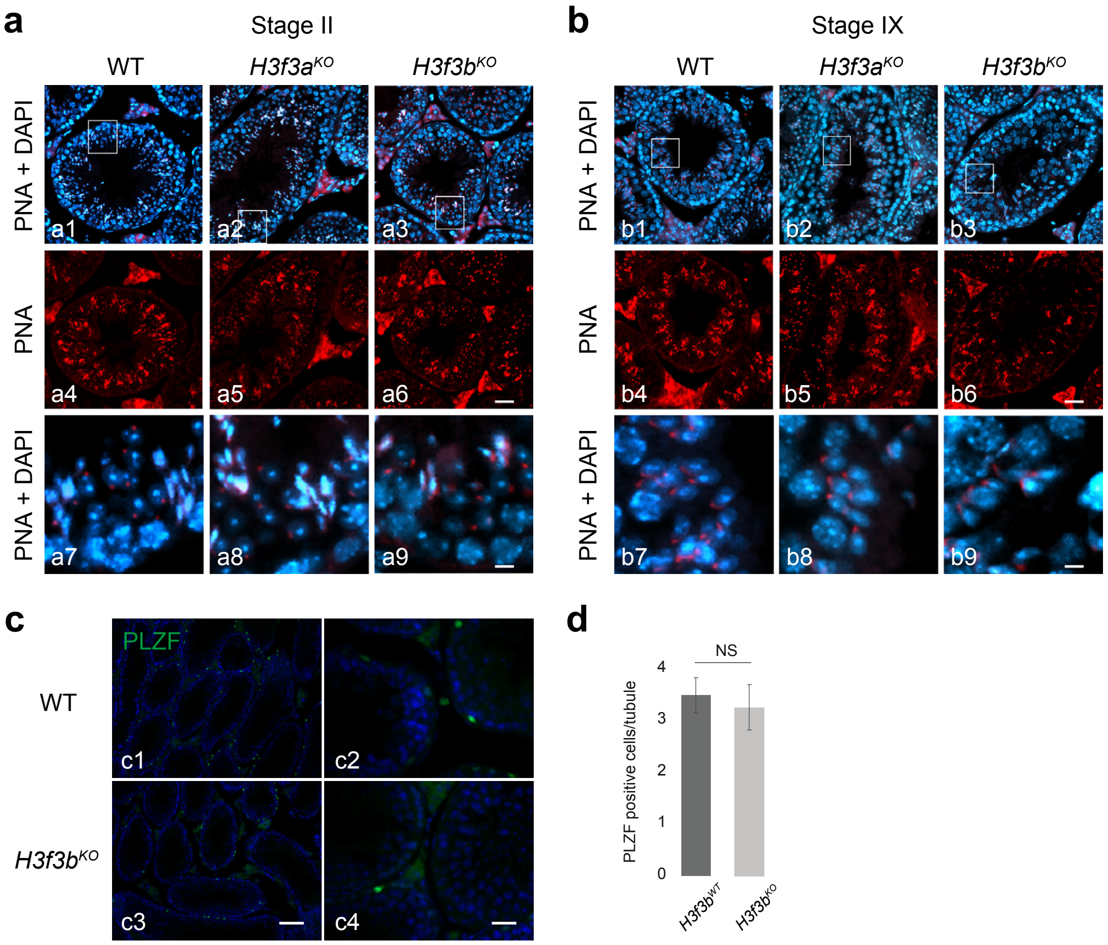


Supplementary Figure 2

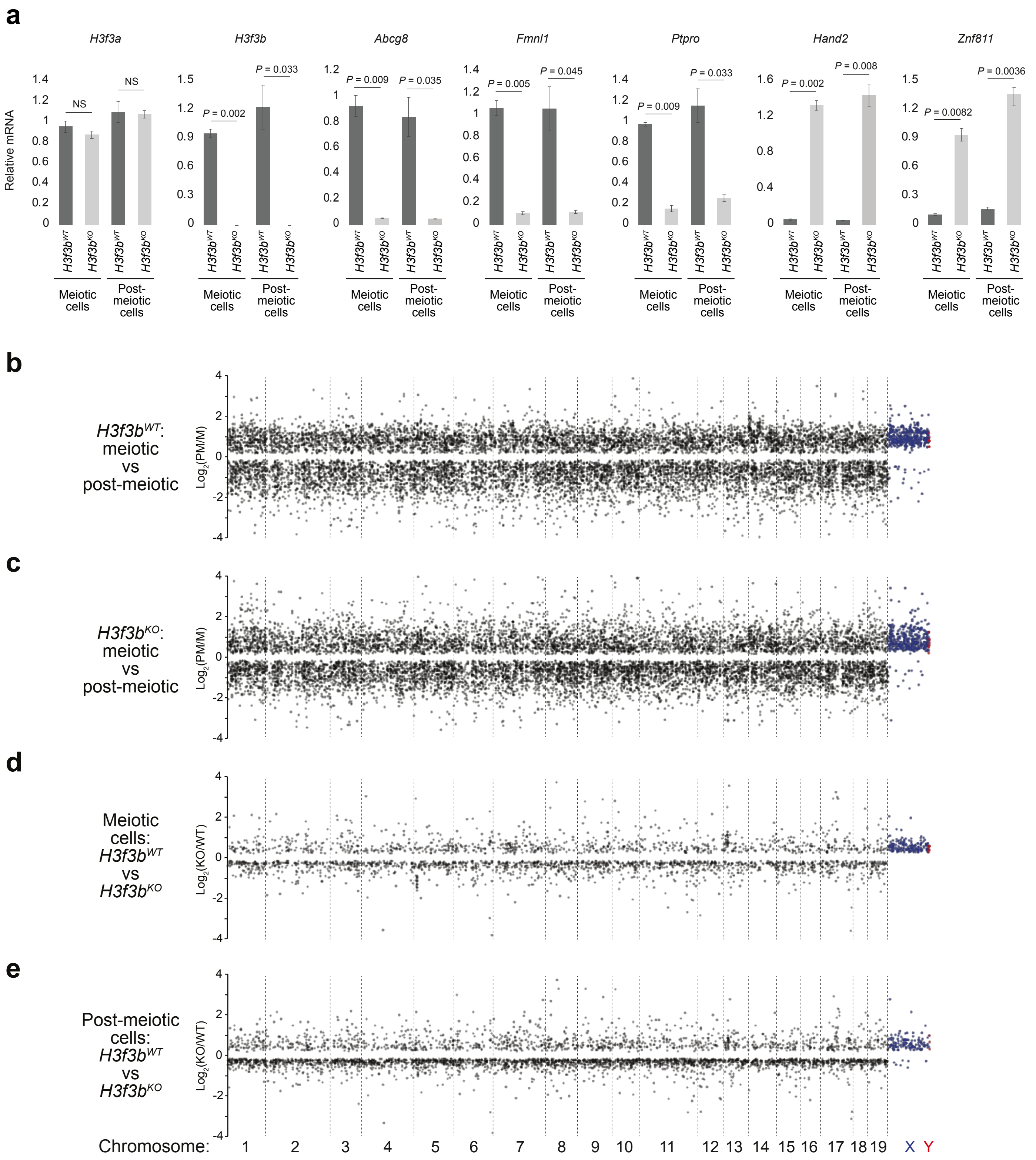


Supplementary Figure 3

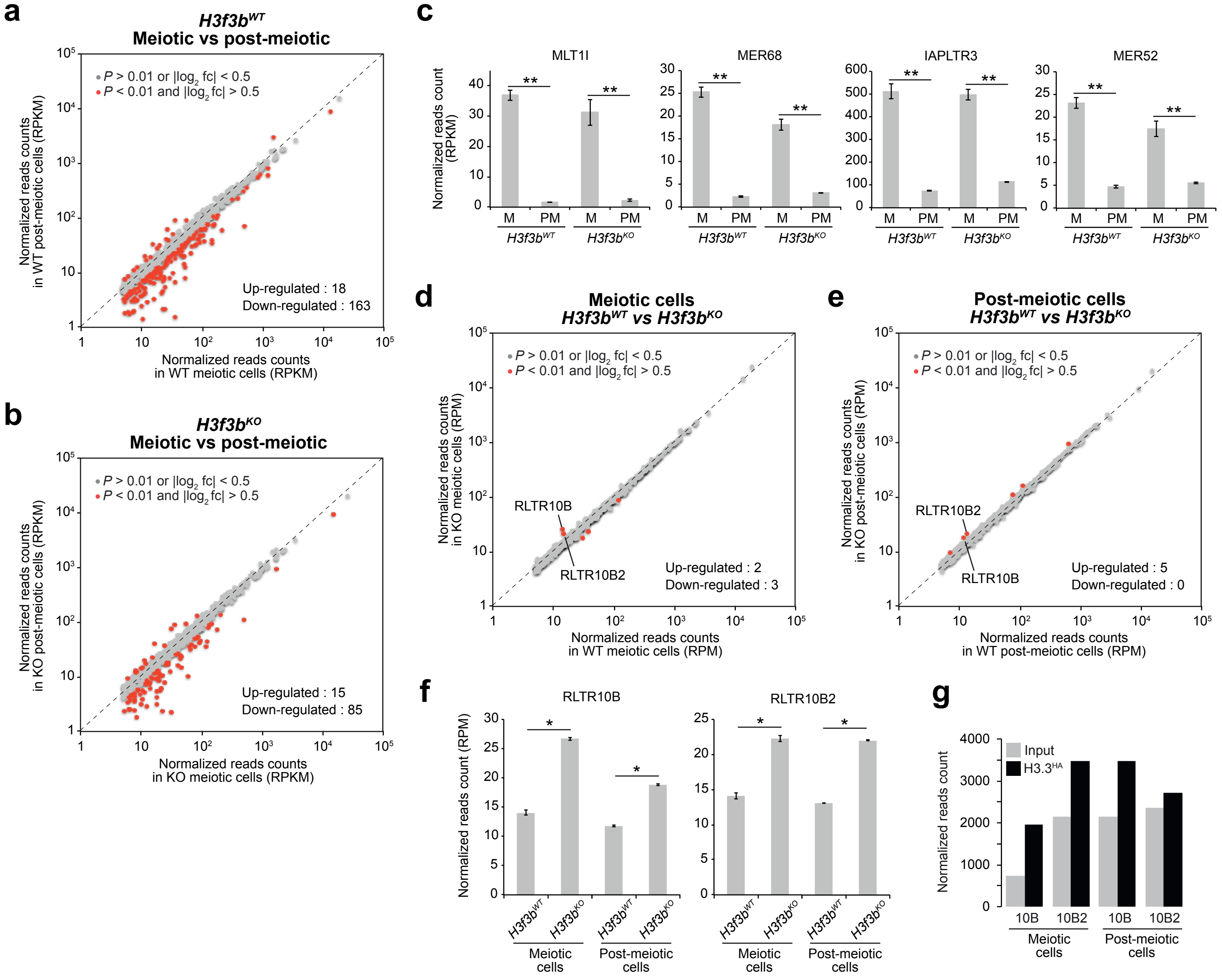


Supplementary Figure 4

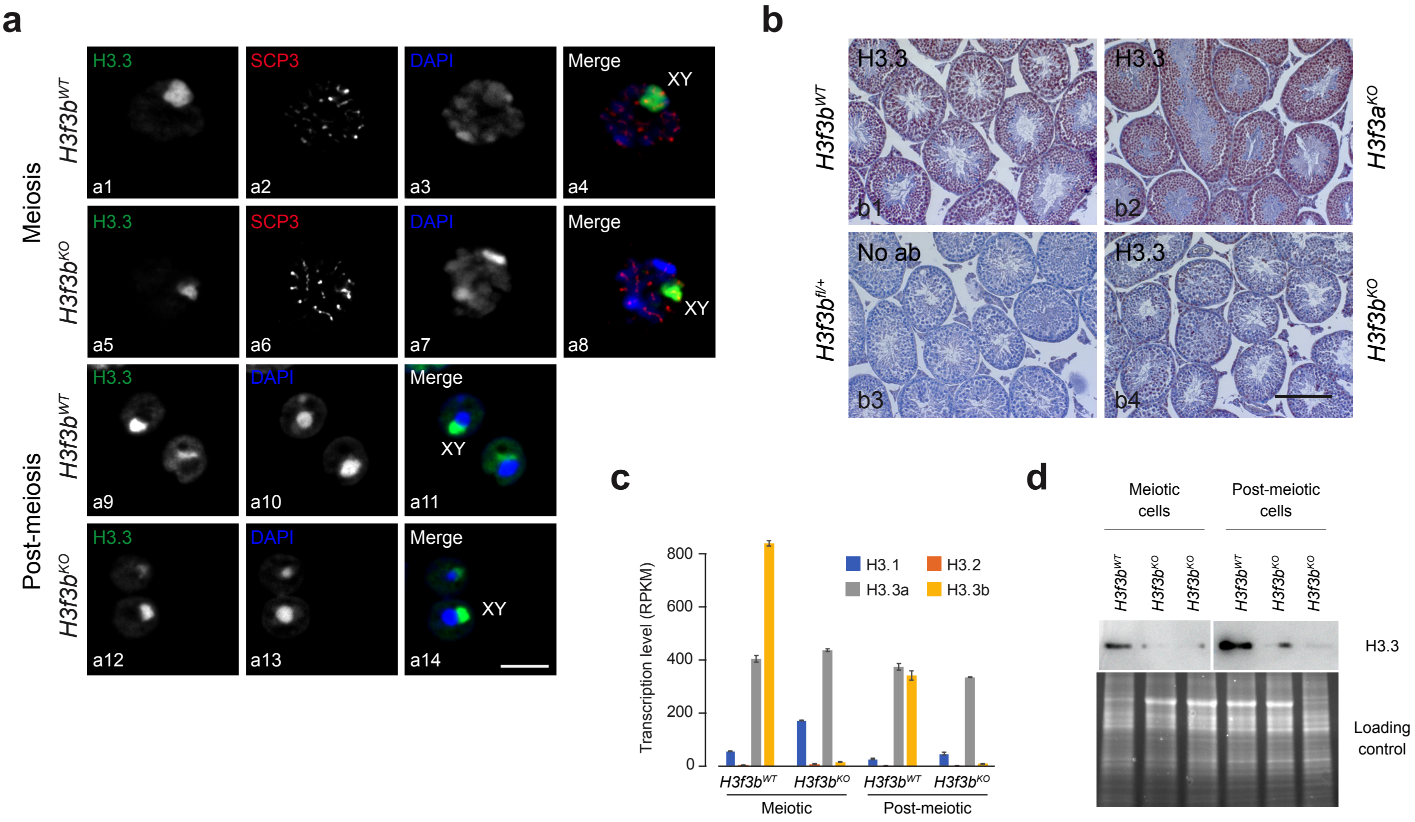


# Supplementary Figure 5

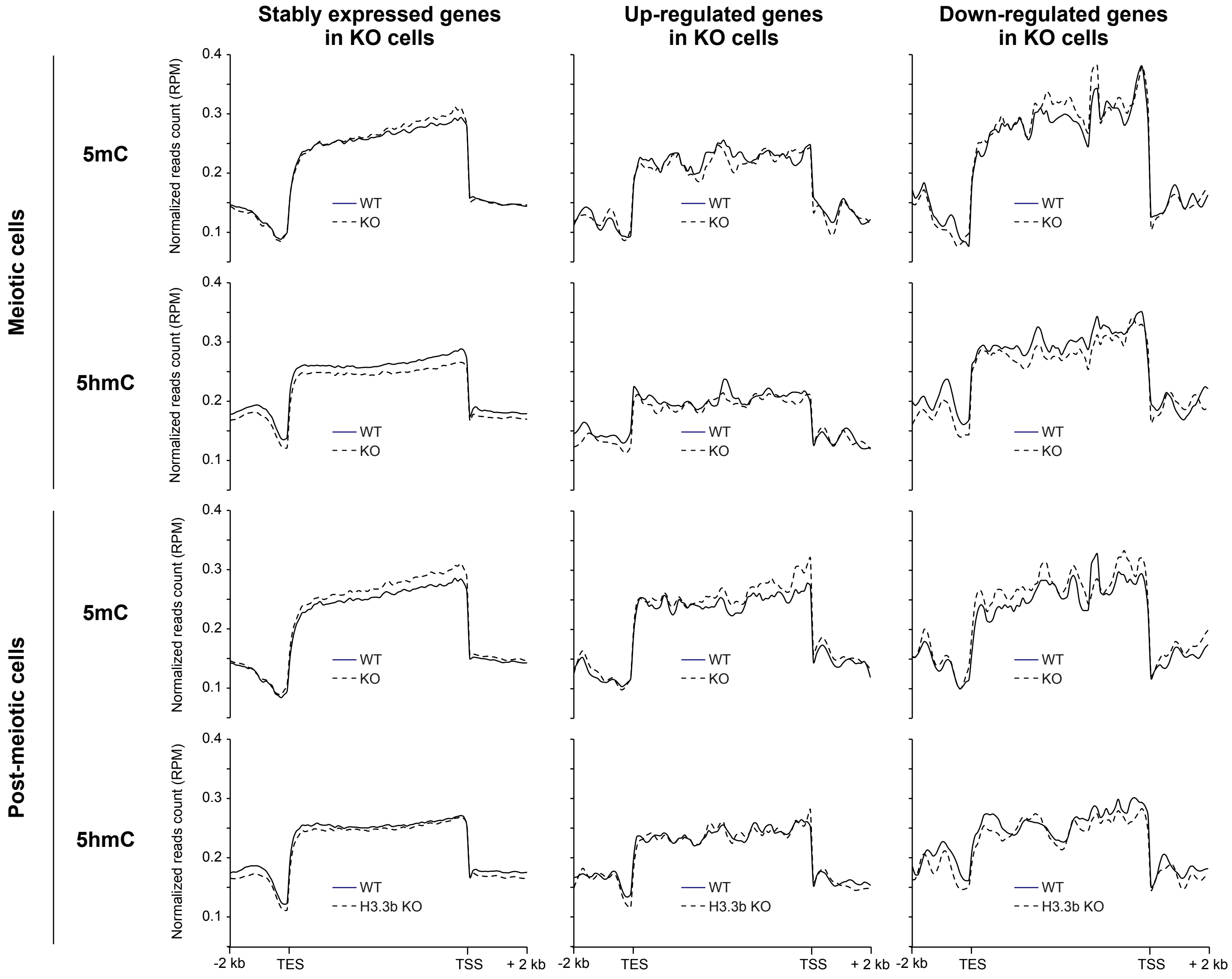


Supplementary Figure 6

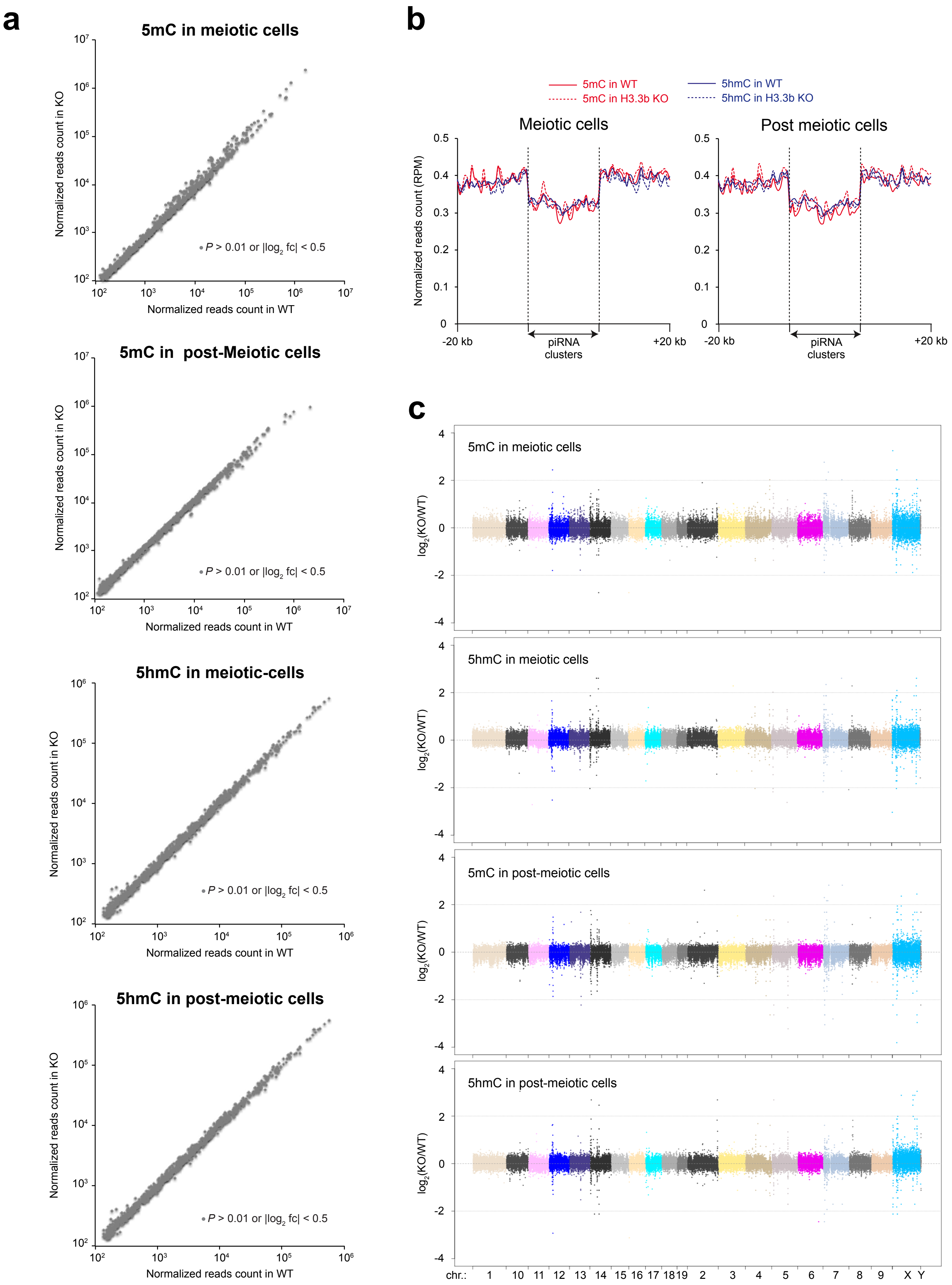


Supplementary Figure 7
